# Automatic Prediction of Peak Optical Absorption Wavelengths in Molecules Using Convolutional Neural Networks

**DOI:** 10.1021/acs.jcim.3c01792

**Published:** 2024-02-29

**Authors:** Son Gyo Jung, Guwon Jung, Jacqueline M. Cole

**Affiliations:** † Cavendish Laboratory, Department of Physics, University of Cambridge, J. J. Thomson Avenue, Cambridge CB3 0HE, U.K.; ‡ ISIS Neutron and Muon Source, STFC Rutherford Appleton Laboratory, Harwell Science and Innovation Campus, Didcot, Oxfordshire OX11 0QX, U.K.; § Research Complex at Harwell, Rutherford Appleton Laboratory, Harwell Science and Innovation Campus, Didcot, Oxfordshire OX11 0FA, U.K.; ∥ Scientific Computing Department, STFC Rutherford Appleton Laboratory, Harwell Science and Innovation Campus, Didcot, Oxfordshire OX11 0QX, U.K.

## Abstract

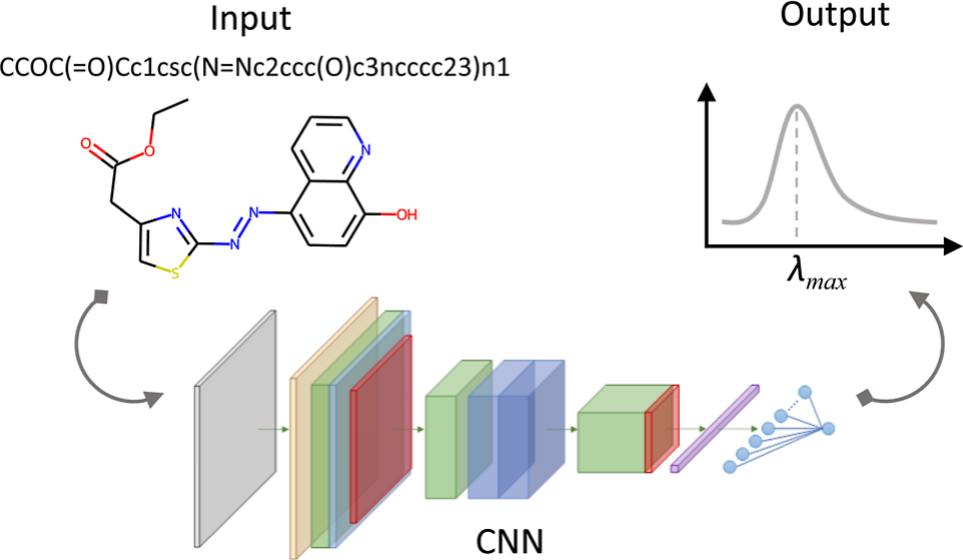

Molecular design depends heavily on optical properties for applications such as solar cells and polymer-based batteries. Accurate prediction of these properties is essential, and multiple predictive methods exist, from *ab initio* to data-driven techniques. Although theoretical methods, such as time-dependent density functional theory (TD-DFT) calculations, have well-established physical relevance and are among the most popular methods in computational physics and chemistry, they exhibit errors that are inherent in their approximate nature. These high-throughput electronic structure calculations also incur a substantial computational cost. With the emergence of big-data initiatives, cost-effective, data-driven methods have gained traction, although their usability is highly contingent on the degree of data quality and sparsity. In this study, we present a workflow that employs deep residual convolutional neural networks (DR-CNN) and gradient boosting feature selection to predict peak optical absorption wavelengths (λ_max_) exclusively from SMILES representations of dye molecules and solvents; one would normally measure λ_max_ using UV–vis absorption spectroscopy. We use a multifidelity modeling approach, integrating 34,893 DFT calculations and 26,395 experimentally derived λ_max_ data, to deliver more accurate predictions via a Bayesian-optimized gradient boosting machine. Our approach is benchmarked against the state of the art that is reported in the scientific literature; results demonstrate that learnt representations via a DR-CNN workflow that is integrated with other machine learning methods can accelerate the design of molecules for specific optical characteristics.

## 1 Introduction

The analysis of optical properties of chemical molecules is pivotal to the molecular design of many applications, including solar cells and polymer-based batteries.^1−6^ An understanding of their absorption and emission characteristics and the effect of external variables on their spectra, such as when injecting current or charged ions into their chemical structure, allows the development of their chemical profile to determine their suitability for certain applications. While there are well-established legacy computational methods to predict optical properties, they are often inaccurate, showing poor agreement with experimental measurements. This discrepancy arises from inherent errors that stem from their approximate nature. These make such methods unsuitable for predicting the optical properties of diverse sets of molecules, particularly when coupled with the considerable computational resources they tend to demand.

The various methods that predict optical properties of chemical molecules include a variety of *ab initio* and data-driven approaches. Theoretical methods, such as high-throughput electronic structure calculations based on time-dependent density functional theory (TD-DFT), have played a vital role in accelerating the discovery of novel chemical materials in many areas of optical and optoelectronic research.^7−13^ Over the last few decades, these methods have become a preferred alternative or complement to experimental research, which is primarily conducted on a trial-and-error basis, which often incurs substantial overhead costs. The process of material characterization, to a certain extent, has been streamlined via *ab initio* methods as they allow for the computational simulation of materials and their properties, with the ability to incorporate continuum solvent approximations.^14,15^ This has enabled an exploration of the vast chemical and property space within various research domains that has proceeded at a pace that cannot be realized via experimentally forged design-to-device workflows.

These efforts within computational materials science have led to the creation of repositories with extensive data sets of chemical structures and properties. The availability of such aggregated chemical information, together with the emergence of big-data initiatives, has led to data-driven approaches surging in interest due to their ability to process and analyze high-dimensional data sets from which previously unseen patterns and relations can be deduced. Machine learning (ML) studies have overhauled various computational techniques by predicting material properties and structures,^16−18^ including those related to the electronically excited states of molecules among others.^19,20^ Such approaches afford lower computational costs compared to high-throughput electronic structure calculations. This exemplifies the effectiveness of ML-based material screening methods for the realization of novel materials within highly complex chemical spaces for various materials science applications.

Although statistical models can be trained to predict ultraviolet–visible (UV–vis) spectra from molecular structural information with relatively lower computational requirements, there are stumbling blocks associated with a typical ML workflow.^21^ These comprise (i) the degree of data sparsity and (ii) the generalizability of molecular representation (for both dyes and solvents). The scarcity of large UV–vis data sets often limits research efforts to typically focus on a subset of the chemical space, such as a single family of dye molecules, and models commonly disregard the solvent environment of the molecules. Recently published open-source data sets of experimental UV–vis spectroscopic properties and computed data sets of excitation energies have helped tackle the data sparsity and chemical diversity issues to a certain extent.^22−34^ Despite such progress, understanding the impact of limited chemical diversity within a training set on model performance is still an important question to address. This is particularly true when analyzing dye molecules from a different part of chemical space to that of the training set, and when trying to incorporate the effect of their solvent environment, which adds an extra layer of complexity to the development of chemical features or representations that are both generic and scalable.

There are notable studies that apply ML techniques, using relatively large training data sets, to predict the peak optical absorption and emission wavelengths or the electronic transition energy of chemical molecules. For example, Ju et al.^25^ employed gradient-boosted regression trees (GBRTs) to predict emission wavelengths and photoluminescence quantum yields using a comprehensive set of experimental data from the literature comprising 4300 samples, of which 3000 are distinct compounds. They leveraged the concept of multiple fingerprint features to combine a number of descriptors, which led to a final input feature vector that is 2741 bits in length. This involved concatenating two circular fingerprints (chemistry development kit (CDK)-extended fingerprints^35,36^ and Morgan fingerprints^37^) with E-state and substructure fingerprints.^38^

In another study, Kang et al.^39^ employed a random-forest regression (i.e., an ensemble learning technique) to predict the excitation energies and associated oscillator strengths of a molecule. They utilized a subset of approximately half a million molecules from the PubChemQC database, which contains TD-DFT calculations of approximately 4 million molecules in PubChem.^34,40−42^ The regression analysis was applied on one-dimensional (1D) and two-dimensional (2D) molecular fingerprints that were generated from SMILES^43^ strings using RDKit;^44^ three-dimensional molecular features were not considered owing to their high computational requirements.

Meanwhile, Joung et al.^45^ employed graph convolutional neural networks (GCNNs), a deep learning approach that is a message-passing neural network whose network structure is defined by the molecular structure,^46,47^ to predict numerous optical properties such as peak absorption and emission wavelengths. This was achieved using an experimental database of 30,094 chromophore–solvent combinations, of which there were 11,392 organic chromophores in 369 different solvents or in the solid state. Each node in a graph network represents an atom, and the corresponding feature matrix comprises the properties of the atom. These include the type of element, number of hydrogens, aromaticity, and hybridization, among others. A graph convolution of the adjacency matrix and the feature matrix updates the atom’s features, leading to a new feature matrix. After a predefined number of iterations, a final feature matrix is reduced to a row vector by summing all elements in order to secure permutation invariance.

More recently, Greenman et al.^48^ employed the open-source Chemprop directed message passing neural network (D-MPNN) framework^49^ to generate fingerprint embeddings to represent dye molecules and solvents for the prediction of molecular absorption peaks. They trained two Chemprop D-MPNN models. The first is trained on 28,772 TD-DFT calculations to predict the TD-DFT peak vertical excitation energy, which is subsequently added to the Chemprop fingerprint embeddings of the second model that is trained to predict the experimentally determined peak absorption wavelength. The study considered 28,734 experimental measurements from several open-source UV–vis data repositories, of which there were 15,157 unique dye molecules and 364 unique solvents, resulting in 26,623 unique dye–solvent pair combinations. Among the previous ML efforts that predict optical properties, only the work of Greenman et al. has utilized the aforementioned multifidelity modeling approach. This allowed them to maximize the accuracy of a model estimate, while minimizing the cost associated with data augmentation through leveraging the vast wealth of computational data that are readily available. Successful demonstrations of such an approach have been shown in other areas of research.^50,51^ It is important to note that, unlike ML-based material property predictions, theoretical-based quantum-mechanical calculations do not suffer from any constraints nor require a priori that is defined by the training data. Therefore, the use of multifidelity data can be advantageous when exploring a chemical landscape that may differ from the training set.

In this paper, we propose a deep learning method that generates learned representations of dye molecules and their cognate solvents using deep residual convolutional neural networks (DR-CNNs), where the inputs to the method are feature matrices that are created exclusively from SMILES strings. We adopt a multifidelity modeling approach to train a ML model on experimental measurements, whose predictive accuracy is improved by auxiliary ML models that have been derived using computational calculations of peak vertical excitation energies in an optical spectrum. Simultaneously, we employ a gradient-boosted and statistical feature selection (GBFS) workflow for material property predictions^17^ in order to identify descriptor features which afford minimal feature redundancy and maximal relevance to the target variable. The incorporation of such a workflow further reinforces the predictability of the final predictive model that is based on the Bayesian-optimized gradient-boosting algorithm. The proposed methods are benchmarked against the state-of-the-art methods that are reported in the scientific literature, and we demonstrate the efficacy and generalizability of the aforementioned methods by applying them on several of the most extensive open-source experimental data sets using two splitting strategies.

Both the GBFS and DR-CNN workflows are designed for general purpose use, emphasizing their versatility beyond the confines of this study. Our paper on the GBFS workflow^17^ showcased its adaptability by predicting various material properties through the fine-tuning of selected features or representations to different target variables using the same workflow. A comparable adaptability is inherent in DR-CNN, designed to generate deep feature representations for a given SMILES string. The overarching nature of our approach, geared toward general applicability, allows us to employ our workflows to a broad spectrum of properties across diverse projects. This encompasses the scope of the present study, whereby these representations are fine-tuned for the UV–vis spectroscopic property of interest, enabling the prediction of optical absorption peaks against their experimental measurements.

## 2 Methods

### 2.1 Workflow Overview

**Figure 1:**
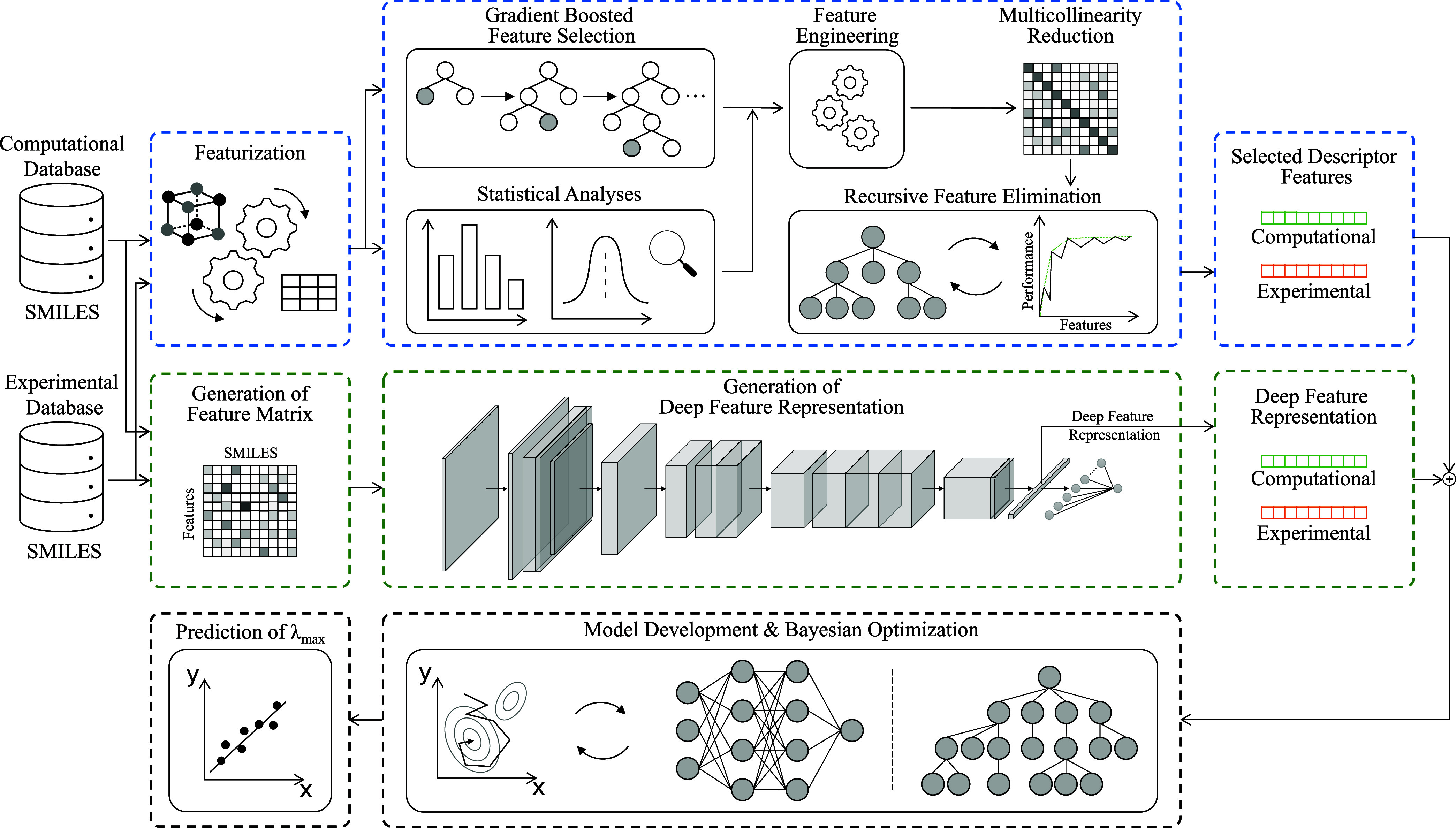
Overview of our operational workflow compartmentalized into two distinct subworkflows. The subworkflow compartmentalization in blue indicates the GBFS framework that has been designed to identify a subset of features which affords minimal feature redundancy and maximal relevance to the target variable.^17^ The subworkflow encapsulated in green shows the deep learning method. This includes feature matrix generation using SMILES strings, followed by the generation of their DFR via DR-CNNs. The selected subset of descriptor features and DFRs are concatenated, and they serve as inputs to the final predictive ML model. The last stage is the development and optimization of the final predictive ML model via Bayesian optimization using Gaussian processes. A portion of the figure has been reproduced with permission from ref (17) [2023].

**Figure 2:**
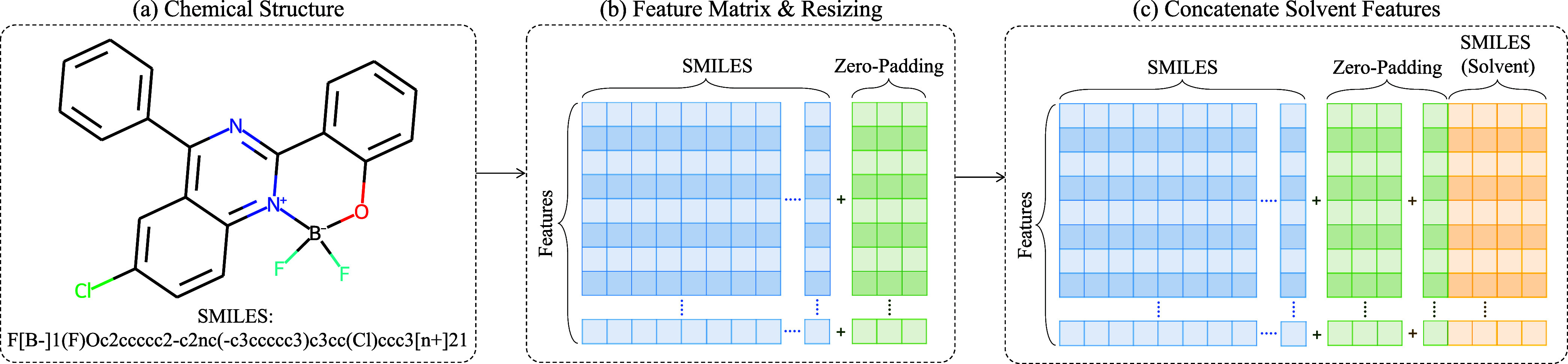
Diagram depicting the process of feature matrix generation from a SMILES string of a chemical molecule. (a) SMILES string is broken up into a sequence of strings using a rule-based tokenizer. (b) Individual symbols, elements, and ions of the chemical molecule form the columns of the feature matrix. The rows of the matrix are subsequently populated when the features defined in Table 2 are detected. The matrix is resized to the required dimensions by applying zero-padding as illustrated. (c) To incorporate the information on a solvent, further zero-padding is applied, and the tokenized SMILES string of the solvent is used to create additional columns to the feature matrix. The rows are populated via the same process as described for (b).

The operational workflow used in this study is shown in Figure 1. There are two distinct subworkflows. These include (i) the GBFS workflow based on our previous work^17^ that has been configured to identify a subset of features which affords minimal feature redundancy and maximal relevance to TD-DFT-calculated peak vertical optical excitation energies and (ii) the deep learning method using DR-CNNs to generate a deep feature representation (DFR) of dye molecules and their associated solvents for the prediction of molecular absorption maxima in optical spectra. The feature matrix that comprises the input to the DR-CNNs is generated solely from the SMILES strings of the dye molecules and their associated solvents, as illustrated in Figure 2.

The aforementioned subworkflows are utilized to process both TD-DFT and experimental data simultaneously. We adopt a multifidelity approach, whereby four models are initially generated in parallel: two from computational data and two from experimental data; these are eventually consolidated into one final ML-based predictive model. On the one hand, the auxiliary DR-CNN outputs a DFT-aware DFR, while an auxiliary GBFS model identifies a subset of selected descriptor features that achieve the maximal loss reduction in predicting the TD-DFT peak vertical optical excitation energies. On the other hand, additional DR-CNN and GBFS models are trained using experimental measurements, and they output an experiment-aware DFR as well as a subset of selected descriptor features that realize the maximal loss reduction; this maximizes their relevance to experimental measurements of the optical absorption peaks. For a given SMILES string, the output of the four models are concatenated. This is fed into the final predictive model that is based on a gradient-boosting algorithm and is Bayesian-optimized using Gaussian processes.^52,53^ Note that a different ML algorithm can be used as the predictive model. In this study, we employed the light gradient-boosting machine, originally developed by Microsoft.^54^

The training and optimization of our models were conducted in the energy domain. This choice aimed to alleviate nonlinear artifacts, particularly in error calculations, and to avoid the reported performance from exhibiting nonlinearity within the spectral domain considered herein. Performing the model training in the wavelength domain would entail assigning equal importance to both small and large errors in energy, contradicting the intended functionality of an objective function. While this practice is marginally acceptable for spectra that absorb red light with errors ranging from ca. 50 to 100 meV, it is notably inadequate for spectra that absorb blue light, where errors surpass ca. 0.5 eV. This disparity in the magnitude of the error has substantial consequences during model training, hence the decision to train in the energy domain. Nevertheless, we present most of the results in wavelength units, as it is the preferred unit for expressing UV–vis absorption spectra among chemists and spectroscopists. Additionally, by reporting in wavelength, we can benchmark our performance directly against relevant studies in the literature, where results are often quoted in wavelength units.

### 2.2 Data Sources, Acquisition, and Splitting

To adopt a multifidelity modeling approach, both experimental and computational data were acquired. The experimental UV–vis absorption data used herein were aggregated from five extensive open-source data tools, sources, or repositories. These included (i) ChemDataExtractor,^23^ (ii) Dye Aggregation,^24^ (iii) ChemFluor,^25^ (iv) Deep4Chem,^26^ and (v) the Dye-Sensitized Solar Cell Database (DSSCDB).^28^ These data sets were chosen due to their size and formatting style. Each chemical data entry contains a SMILES string of a dye molecule, and this was accompanied by the peak optical absorption wavelength and solvent information. Not every solvent in these data records was presented in SMILES format. When such a SMILES string was unavailable, it was acquired from the data set complied by Greenman et al., who identified the corresponding SMILES via the mapping between solvent information and a manually curated dictionary of SMILES strings. The data set was manipulated via the filtration of valid dye and solvent SMILES strings using RDKit,^44^ in addition to the elimination of entries with multiple dye molecules or molecular clusters. This resulted in a data set that comprised 28,734 sets of experimental measurements as summarized in Table 1; this is consistent with the data in ref (48). Further data cleaning was undertaken to deal with duplicate entries and inconsistent wavelength measurements at a tolerance level of 5 nm. The ChemDataExtractor-generated data set was put aside from the combined experimental data set. This was in order to achieve a like-to-like comparison to a state of the art in the literature, namely, the work of Greenman et al., as they did not use the ChemDataExtractor-generated data set. This data-removal process led to the remaining 26,395 records.

**Table 1:** List of Data Sources and the Corresponding Number of Experimental Measurement of Peak Optical Absorption Wavelengths^a^

data tool or source	number of entries
ChemDataExtractor	1915
Dye Aggregation	3626
ChemFluor	4170
Deep4Chem	16,585
DSSCDB	2438
total	28,734

a This table summarizes the number of data entries after filtering; it does not reflect the entire data entries from each data tool or source and the duplication that exists between them.

The computational data used in this study were obtained from Beard et al.^23^ and Greenman et al.^48^ The data set provided by Beard et al. were realized via high-throughput electronic structure calculations that employed DFT within the simplified Tamm–Dancoff approximation (sTDA-DFT) and traditional TD-DFT. Chemicals were selected for these calculations where experimental data about their optical absorption wavelength had been mined from scientific literature using the “chemistry-aware” text-mining tool ChemDataExtractor.^23,55^ Thereby, the calculated data provided a theoretical counterpoint to the chemically matching experimental data. We used the entire set of 6142 sTDA-DFT calculations for this study.

The computational data set provided by Greenman et al. included 28,772 geometrically optimized TD-DFT calculations. These calculations employed the Tamm–Dancoff approximation (TDA)^56^ ωB97X-D3^57^/def2-SVPD level of theory using the ORCA software.^58^ In preparation for such calculations, initial molecular geometries were generated by using RDKit^44^ to convert SMILES strings into Cartesian coordinates. These initial geometries were refined employing semiempirical tight-binding DFT (GFN2-xTB^59^). These geometries were subsequently optimized at the BP86^60^-D3^61^/def2-SVP^62^ level of theory. Solvent corrections were also employed using the integral equation formalism polarizable continuum model (IEFPCM) in the Gaussian software.^63^ Cognate experimental data in solution exist for 19,409 or 10,409 of these molecular structures that were calculated under vacuum or solution, respectively. We note that 21 SMILES strings of dye molecules were incompatible with certain descriptors used in the GBFS workflow.

The proposed methodology was evaluated using two types of splitting strategies for the experimental measurements. This was to ensure that we conducted a fair assessment of the generalizability of the ML models since the choice of the splitting strategy can sometimes lead to an underestimation of the predicted errors.^64,65^ The strategies adopted were (i) random splitting by dye–solvent pairs and (ii) scaffold splitting using the Bemis–Murcko scaffold framework.^66^ The former approach ensures that there is no overlap of dye–solvent pairings between the training and test sets given the removal of duplicated entries. Such a strategy, however, overlooks the potential correlation between measurements of the same dye in different solvents, which may lead to an overestimation of the model’s predictability. Scaffold splitting addresses the potential overestimation of a model’s performance. Thereby, a Bemis–Murcko scaffold was employed using a constraint that any dye molecules with an identical scaffold are present only in one of the data sets. This ensures that the realized errors are more reflective of the model’s true generalizability.

For both splitting strategies, the split ratio of 9:1 was used for the training and test sets, respectively. 20% of the training set was then retained as an out-of-sample validation set. Moreover, to ensure an unbiased comparative analysis to the state-of-the-art models reported in the literature, the results pertaining to the scaffold splitting strategy presented hereafter are computed using the same data sets that were used in the study by Greenman et al.

### 2.3 Featurization

There are two separate featurization steps that run in parallel; the workflow for each of them is shown in Figure 1. For the featurization step that involves the GBFS workflow, a high-dimensional feature vector was generated by leveraging an extensive set of descriptors that take SMILES as inputs. These included the use of chemical feature descriptors such as Morgan fingerprints, RDKit molecular fingerprints, ElemNet, Maccs keys, element property fingerprints, and atom pair counts.^37,44,67−73^ When using all of these descriptors, a base feature vector (i.e., prefeature engineering) of length 11,173 is created for each material by concatenating the output of these descriptors. The resulting feature vectors serve as inputs to the GBFS workflow, which identifies a subset of features that exhibit maximal loss reduction when predicting the optical absorption peaks. More information on the GBFS workflow is provided in Section 2.4.

For the featurization step that involves a DR-CNN workflow, a 2D feature matrix or image is created from a SMILES string. The idea stems from image-processing methods that use convolutional neural networks, whereby high-dimensional, usually 2D or 3D, images are used to train and generate low-dimensional representations or feature vectors of the original images. These low-dimensional representations are subsequently fed into a fully connected network that has been optimized to perform either a classification or a regression analysis. The application of such a concept to computational chemistry means that low-dimensional representations of chemical molecules can be acquired from their corresponding feature matrices or images. Furthermore, these representations can be optimized to discriminate between target classes or to predict a target variable; this results in context-aware representations, as supposed to a fixed type of feature, that can be computed via standardized chemical or structural feature descriptors.

In this work, we create a feature matrix exclusively from SMILES strings. The SMILES representation is constructed from symbols that represent certain chemical and structural information about a molecule.^43^ A SMILES string is broken up into a sequence of strings using a rule-based tokenizer, and the individual string (i.e., a symbol, element, or ion) forms the columns of the feature matrix, as shown in Figure 2. The resulting matrix is subsequently populated when certain features are detected, where each row represents one of the features listed in Table 2. The feature matrix is resized via a zero-padding operation. In order to incorporate solvent information, further zero-padding is applied, and the tokenized SMILES strings of the solvents in question are used to create additional columns in the feature matrix. The rows of this part of the matrix are populated in a manner similar to the part that is associated with the dye molecule.

**Table 2:** List of Features Used to Generate the Feature Matrix, as Shown in Figure 2^a^

feature type	feature symbol or name	description	length
SMILES	(	start of branch	1
	)	end of branch	1
	[	start of atom group	1
	]	end of atom group	1
	.	ionic bond	1
	:	aromatic bond	1
	=	double bond	1
	#	triple bond	1
	\	cis	1
	/	trans	1
	@	chirality	1
	+	positive charge	1
	-	negative charge	1
	ionic charge	no. of ionic charge (2–7)	6
	ring	start of ring	1
	ring	end of ring	1
chemical	element	H, C, O, N, P, S, or others	7
	no. of H	total no. of hydrogen	1
	degree	degree of unsaturation	1
	charge	formal charge	1
	valence	total valence	1
	ring	within a ring	1
	aromatic	within an aromatic structure	1
	tetrahedral chirality	clockwise, counter-clockwise or unrecognized	3
	hybridization	s, sp, sp2, sp3, sp3d, sp3d2, or unrecognized	7

a The resulting feature matrix is used as an input to the DR-CNNs in Figure 3. These features are computed using RDKit^44^ following the tokenization of the SMILES string.

Each feature matrix that is generated from a SMILES string contains 44 rows or features, as summarized in Table 2. The features are categorized into two types: (i) symbol-based SMILES and (ii) chemical-based information. The rows of the feature matrix are set up to detect for 21 SMILES symbols and 23 chemical-based features, which include element types, number of hydrogen, degree of unsaturation, formal charge, total valence, ring, aromatic, chirality, and hybridization. The four features listed in Table 2 (i.e., the number of ionic charge, type of element, chirality, and hybridization) were treated as categorical features by representing them using one-hot encoding. These features are detected using RDKit,^44^ and additional features can be appended as a user option. It should also be noted that ambiguities can arise for compounds that can be represented in many different SMILES formats. These ambiguities can be eliminated during the preprocessing stage of the DR-CNN workflow, either manually or via the use of a normalization algorithm, to ensure that unique SMILES representations for each chemical compound are supplied as input to the feature matrix generation stage.

Although the number of rows (features) is fixed, the number of columns (symbols) is determined by the longest SMILES string in the data set, by default. However, if solvent representations are disregarded, the user can predetermine the maximum number of columns, while SMILES strings that are shorter than the predetermined limit can be regulated using zero-padding. For studies where solvent representation is of importance, the limit can also be predetermined by considering the limit for the maximum SMILES length of the dye molecule and the solvent as well as the zero-padding between the two representations. In our algorithm, the default limiting number of columns in the feature matrix is set by the combination of the longest SMILES string of the dye molecule, the predetermined zero-padding layers, and the longest SMILES string of the solvent. Predetermining this limit so that the size of the feature matrix is known prior to carrying out any calculations is helpful because its dimensions can be used to optimize the computational cost associated with developing the DR-CNN. Within this scope, it can also minimize the number of zero-padding layers that can be applied.

### 2.4 Gradient Boosted and Statistical Feature Selection Workflow

The GBFS workflow leverages a gradient boosting framework to identify a subset of features that maximizes their relevance to the target variable or class. We defined the relevance of a feature as the total amount of loss reduction that is realized when a split is performed on a leaf node using the feature. That is, the significance of the split points in learning the decision trees is used to determine the relevance of a feature to the target variable or classes; therefore, features that provide optimal tree growth are selected. The selected features are further refined using descriptive statistical analyses and are tested for multicollinearity, by performing correlation and hierarchical cluster analyses, whereby features are eliminated if they exhibit a correlation coefficient above 0.9 or a Ward’s linkage distance below a certain distance threshold, as determined using the elbow method. Subsequently, we identified the optimal subset of features among the candidate features by employing a wrapper method based on recursive feature elimination. This employs a greedy search approach that is used to train an estimator and evaluate a different combination of features against a performance metric in a recursive manner. More details can be found in ref (17).

### 2.5 Deep Residual CNN Architecture

Our DR-CNN architecture takes the 2D feature matrix described in Section 2.3 as input. Its architecture, inspired by deep residual learning,^74^ consists of five stages as shown in Figure 3a. The last four stages each include a convolutional block (ConvBlock) or a combination of a ConvBlock and an identity block (IdentBlock). The architecture of these two types of blocks is depicted in Figure 3b,c, along with operations performed and the number of filters used. In common with the popular ResNet models, skip or residual connections are implemented with nonlinearities (e.g., ReLU) and batch normalization.

**Figure 3:**
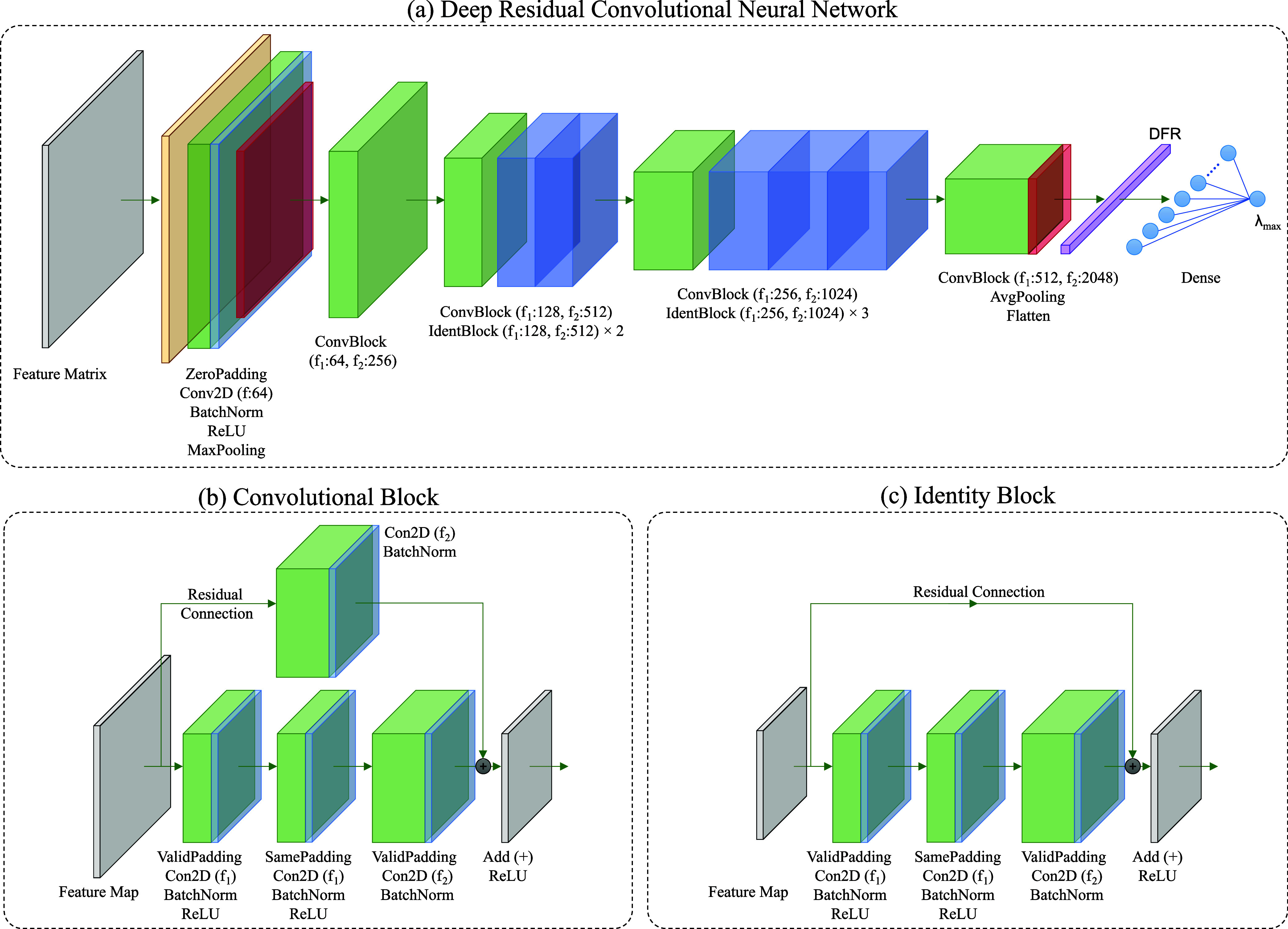
(a) Schematic of the DR-CNN model architecture. As an input, the network takes in a feature matrix, as defined in Figure 2, whose features are listed in Table 2. The first convolutional layer consists of ZeroPadding, Conv2D with 64 filters (i.e., f:64), BatchNorm, ReLU, and MaxPooling operations, as denoted in the diagram. The subsequent stages of the DR-CNN model architecture consist of (i) convolutional block (ConvBlock) with f_1_:64 and f_2_:256; (ii) ConvBlock with f_1_:128 and f_2_:512 and two identity blocks (IdentBlock) each with f_1_:128 and f_2_:512; (iii) ConvBlock with f_1_:256 and f_2_:1024 and three IdentBlock each with f_1_:256 and f_2_:1024; and (iv) ConvBlock with f_1_:512 and f_2_:2048, AvgPooling, and Flatten operations, leading to a deep feature representation (DFR). A DFR feeds into a fully connected layer with a ReLu activation function and an output node that has a linear activation. (b,c) Architecture of the ConvBlock and IdentBlock, respectively, where f_1_ and f_2_ denotes two different filter sizes, as defined in (a).

The effect of network depth is highly influential in pattern or visual recognition tasks, with deep networks having the ability to learn complex patterns within a given data set. However, deep networks are prone to problems that are directly associated with their depth. In particular, the problem of the vanishing or exploding gradient needs to be addressed, as it hinders convergence.^75,76^ Consequently, batch normalization is used to circumvent the convergence issue,^77^ although convergence with batch normalization gives arise to another complication, namely, the degradation problem. The degradation problem manifests itself with increasing network depth as accuracy degrades rapidly from a point of saturation; this is not a result of overfitting. Therefore, in order to benefit from the characteristics of a deep network while minimizing the aforementioned problems, we employ the deep residual learning approach with a residual or skip connection. These residual connections ensure that we do not degrade the model when additional layers are introduced, which, in turn, increases the size of the searchable function space. This is equivalent to nesting the function space such that the model approximation does not move away from the current search space; thereby, guaranteeing that while the model can improve with more layers, it will not do any worse as regularization will skip over them if the addition of layers does not prove to be useful.

The key difference between ConvBlock and IdentBlock is that there is no change in the input and output dimensions of the latter. In ConvBlock, there is a convolutional layer in the residual connection. This changes the dimensions such that the addition operator is applied to two matrices of equal size, and the output matrix has reduced dimensions compared to the input matrix.

It is important to emphasize that similar to the GBFS workflow, DR-CNN is a general purpose workflow. This means that it is not designed specifically for the prediction of molecular absorption peaks; rather, its primary purpose is to generate deep representations of given molecules by incorporating a diverse set of features that are not exclusively tailored to a particular material property. These representations are subsequently fine-tuned against a user-defined target property or variable. Therefore, we do not constrain the selection of features to those directly pertinent to a specific material property as the workflow can be adapted for fine-tuning against other variables. In instances where certain features may be irrelevant to the target, the workflows are designed to handle this by eliminating such features (in GBFS) and either mitigating or disregarding their influence through regularization techniques (in DR-CNN). Indeed, the fact that our workflow automatically eliminates features that are known to be irrelevant to a given property of interest can help in the validation stage of a GBFS-based study; their selective removal demonstrates that our workflow can successfully discriminate features that should be discounted from a scientific standpoint.

### 2.6 Bayesian Optimization Using Gaussian Processes

A two-step optimization process was followed to determine the architecture of the final predictive models that are based on the gradient-boosting algorithm. The hyperparameters of these ML models were optimized using a combination of grid search and Bayesian optimization using Gaussian processes, i.e., a sequential model-based approach. An initial hyperparameter tuning process was performed by scanning the hyperparameter space by using the grid search method. This subsequently identified the region in which Bayesian optimization was to be applied. Such an optimization strategy proves particularly effective for an objective function that has no closed form, is expensive to evaluate, and results in noisy responses.

The Bayesian optimization in this work incorporated three acquisition strategies, and they are (i) Probability of Improvement,^78^ (ii) Expected Improvement,^79^ and (iii) Upper Confidence Bounds.^80^ At each iteration, the three acquisition functions were optimized, and each was made to propose a query point independently. One of the query points was then chosen based on a probability calculation using a softmax function that parametrizes the weights of the gains, which were initially set to zero. Once the surrogate model had been fitted with the new query point, the gains were updated by using the mean evaluated at the new point. See Supporting Information 1 for a discussion on Bayesian optimization and the acquisition schemes used in this study. The corresponding pseudocode can be found in Supporting Information 2.

## 3 Results and Discussion

### 3.1 Efficacy of Auxiliary ML Models: Predicting λ_max_ Values against DFT Data

**Figure 4:**
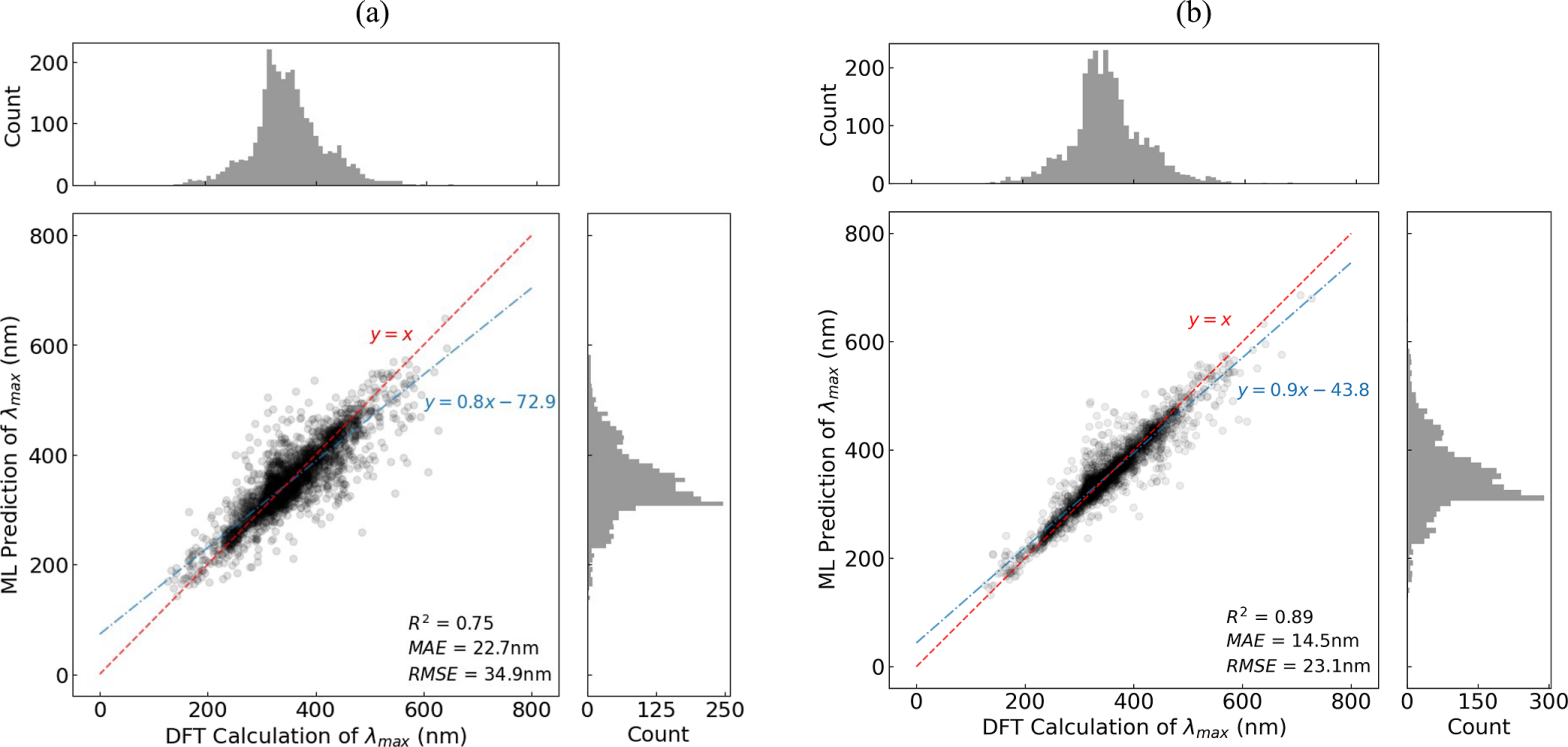
Regression analysis of the ML-based prediction of vertical excitation energies, and their corresponding peak wavelengths (λ_max_), against the DFT-based calculations. The two separate predictions were made by two Bayesian-optimized gradient boosting models that were trained on (a) DFR from the DR-CNN subworkflow and (b) descriptor features from the GBFS subworkflow. The analyses were conducted using the 28,751 vacuum TD-DFT calculations that were sourced from ref (23,48) with a train-to-test split ratio of 9–1. The solid blue line is a linear fit between the DFT- and ML-based predictions, generated using the ordinary least squares. The dashed red line is drawn to represent the hypothetical case, where the ML-based prediction would equal the DFT-based calculations.

The prediction of vertical excitation energies and their corresponding peak wavelengths (λ_*max*_) made by the Bayesian-optimized gradient boosting models on the test set are shown in Figure 4. The inputs to these models were the DFT-aware features generated by either the DR-CNN or the GBFS subworkflows, whose inputs were the aforementioned feature matrices and descriptor features, respectively, that were generated using TD-DFT data described in Section 2.2. The DR-CNN and GBFS subworkflows achieved λ_*max*_ predictions with a mean absolute error (MAE) of 22.7 and 14.5 nm, a root-mean-square error (RMSE) of 34.9 and 23.1 nm, and a coefficient of determination (*R*^2^) of 0.75 and 0.89 on the out-of-sample test set, respectively. The equivalent regression analyses of the DR-CNN and GBFS-based predictions of λ_max_ using the sTDA-DFT data set led to a test set MAE of 22.8 and 14.1 nm, an RMSE of 31.2 and 23.3 nm, and an *R*^2^ of 0.71 and 0.88, respectively. The regression details associated with these model predictions can be found in Supporting Information 3.

**Figure 5:**
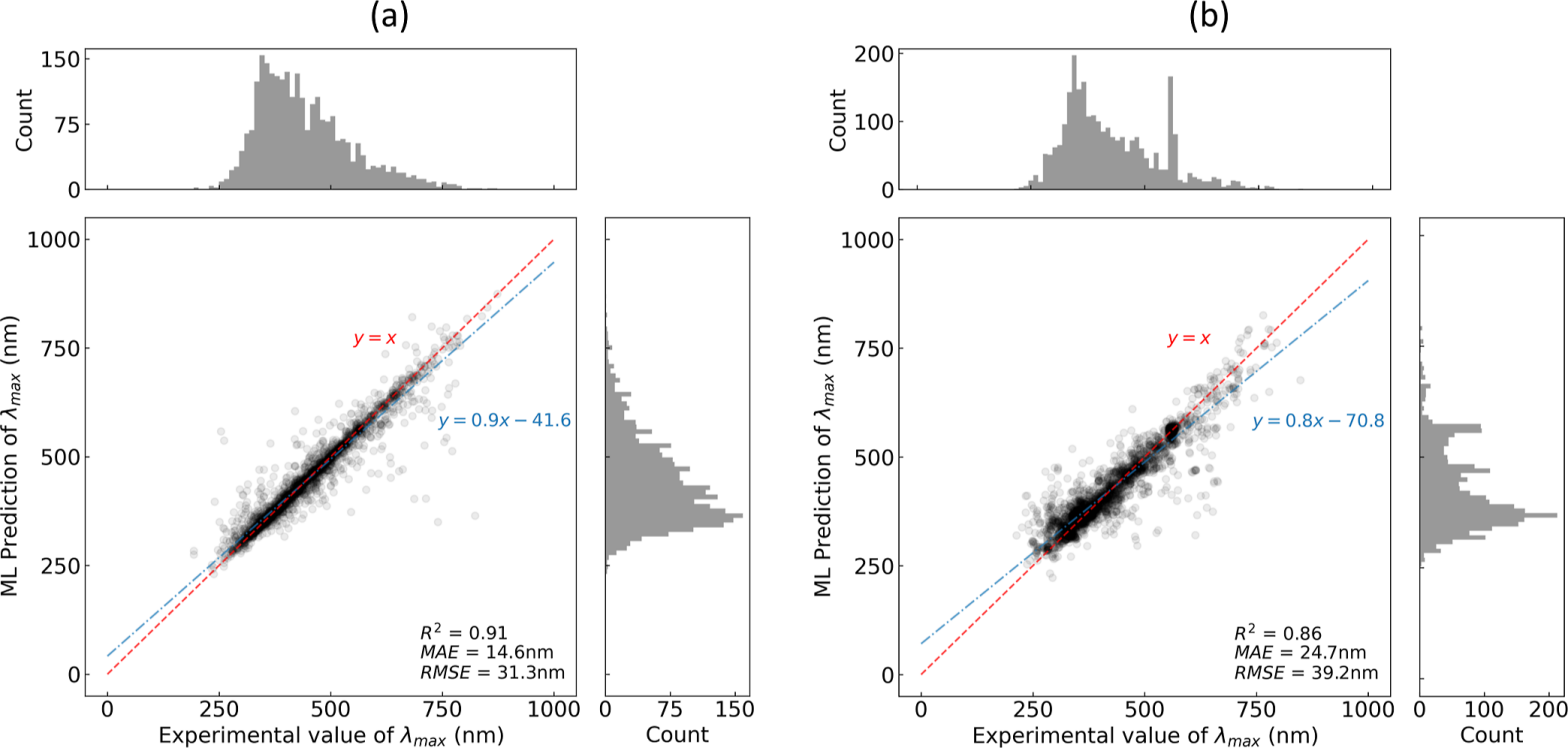
Regression analysis of the multifidelity ML model prediction of the optical absorption peak wavelength (λ_max_) values of unseen chemical dyes against their experimental measurements. The two separate predictions were made by two Bayesian-optimized gradient boosting models with a training set that were afforded using (a) random and (b) scaffold splitting strategies, respectively. The solid blue line is a linear fit between the experimental measurements and ML-based predictions, generated using ordinary least-squares refinement. The dashed red line is drawn to represent the hypothetical case, where the ML-based prediction would equal the experimental measurements.

**Figure 6:**
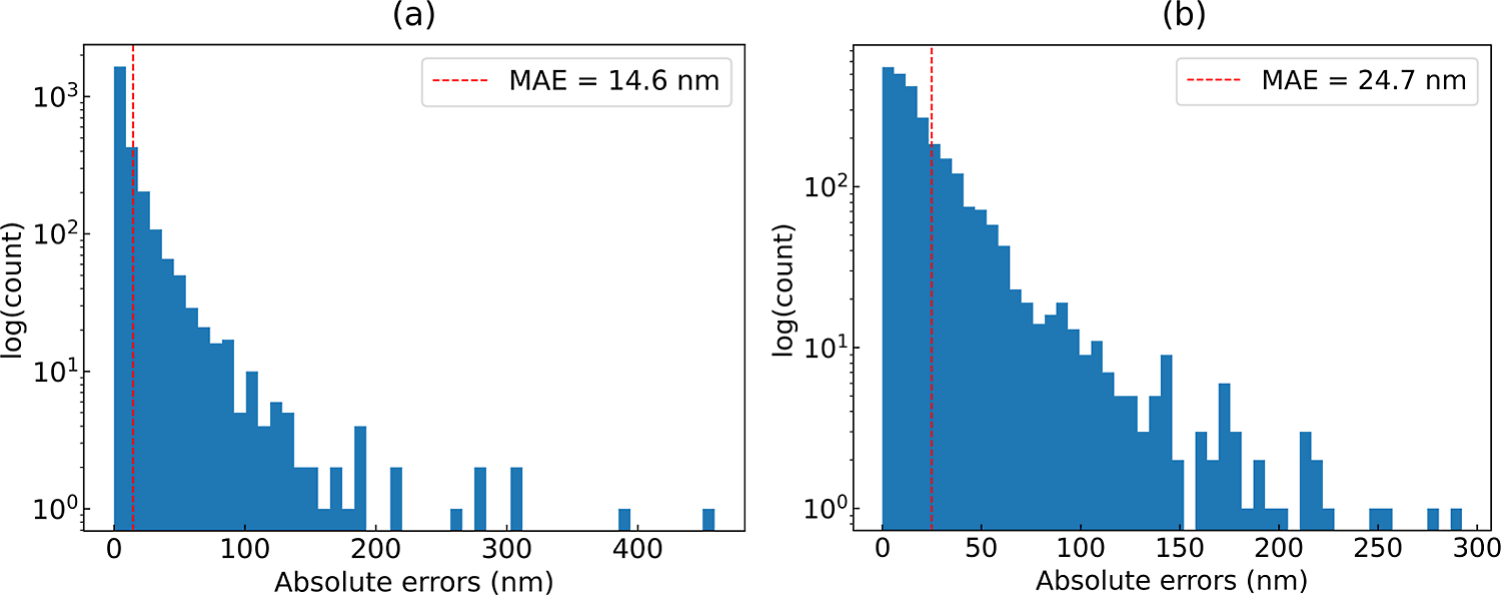
Distributions of the absolute errors of the ML-based prediction of the optical absorption peak wavelength (λ_max_) against the experimental measurements. (a) Error distribution obtained using the random splitting strategy and (b) error distribution obtained using the scaffold splitting approach. The dashed red line indicates the MAE.

For this part of the analysis, we did not concatenate the outputs of the two subworkflows to develop a single predictive model for each of the DFT data sets. The rationale was to perform separate regression analyses in order to examine the difference in their performance levels. We observed that lower errors were realized when predictions of λ_max_ values were made using the GBFS workflow. However, certain descriptors failed to process some of the SMILES strings, while the generation of feature matrices for the DR-CNNs did not suffer from such an issue.

Moreover, we adopted a random splitting strategy to train the DFT-based ML predictions of λ_max_ values with a rationale that the development of the ML-based methods is decoupled from the process of DFT-based calculations. Indeed, the computational data were readily available prior to designing a framework to predict the experimental optical absorption peak wavelength of unseen chemical dyes.

### 3.2 Efficacy of Multifidelity ML Models: Predicting λ_max_ Values against DFT Calculations and Experimental Measurements

The combined data set of experimentally measured λ_*max*_ values was passed through the operational workflow of our system architecture, whereby the DR-CNN and GBFS models were trained to optimize a λ_*max*_ prediction model. By applying the test set of these data to our method with the random splitting strategy, our method realized an MAE of 16.5 and 23.3 nm, an RMSE of 33.1 and 44.4 nm, and an R^2^ of 0.90 and 0.82, using the GBFS and DR-CNN subworkflows, respectively. When the scaffold splitting strategy was used to create the test set, our method achieved an MAE of 28.2 and 35.9 nm, an RMSE of 41.2 and 49.5 nm, and an R^2^ of 0.84 and 0.77, through the GBFS and DR-CNN subworkflows, respectively. The realized errors and the coefficient of determination for each subworkflow and splitting strategy are summarized in Table 3. The corresponding regression plots can be found in Supporting Information 4.

**Table 3:** Summary of the Model Performance Categorized by the Choice of Workflow, Data Type, Splitting Strategy, and the Statistical figures of Merit, which Includes MAE, MSE, and the Coefficient of Determinant (*R*^2^)

data	workflow	split strategy	MAE (nm)	RMSE (nm)	*R*^2^
sTDA-DFT	GBFS	random	14.1	23.3	0.88
	DRCNN	random	22.8	31.2	0.71
TD-DFT	GBFS	random	14.5	23.1	0.89
	DRCNN	random	22.7	34.9	0.75
experimental	GBFS	random	16.2	32.2	0.91
	DRCNN	random	23.3	44.4	0.82
	GBFS	scaffold	27.0	39.3	0.85
	DRCNN	scaffold	35.9	49.5	0.77
	multi-fidelity	random	14.6	31.3	0.91
		scaffold	24.7	39.2	0.86

**Table 4:** List of Features Identified to Have the Most Relevance in the Prediction of λ_max_ Values against Their Experimental Measurements of Optical Absorption Peak Wavelength^a^

no	feature abbreviation	feature description
1	DR-CNN TD-DFT DFR-345	DR-CNN DFR of TD-DFT (index 345)
2	DR-CNN TD-DFT Peak	DR-CNN-based TD-DFT λ_max_
3	GBFS sTDA-DFT Peak	GBFS-based sTDA-DFT λ_max_
4	MACCSkeys-49	MACCS keys 49 (i.e., C = C(C)C)
5	NumAliphaticRings	no. of aliphatic rings
6	MaxAbsEStateIndex	maximum absolute E-state index
7	MorganFeature3Counts	count-based Morgan fingerprint features of radius 3 (index 31)
8	RDKitFPBits-1285	RDKit fingerprint bits (index 1285)
9	Fr_Allylic_Oxid	no. of allylic oxidation sites (excl. steroid dienone)
10	MaxEStateIndex	maximum E-state index
11	SlogP_VSA1	MOE-type descriptor using log of the octanol−water partition coefficient and van der Waals surface area contributions (index 1)
12	LabuteASA	labute accessible surface area value
13	BCUT2D_MWLOW	2D Burden eigenvalue (low mass eigenvalue)
14	PEOE_VSA8	MOE-type descriptor using partial charges and van der Waals surface area contributions (index 8)
15	VSA_EState2	MOE-type descriptor using E-State indices and van der Waals surface area contributions (index 2)

a See Figure 7 for the corresponding total loss reduction realized by the features.

These results reflect the effectiveness of the experimentally aware DFR and feature descriptors in the prediction of the optical absorption peak. We subsequently concatenated the two representations along with those generated by the auxiliary models, to afford the input to the final gradient boosting model that was refined by Bayesian optimization. Our multifidelity ML model realized an MAE, an RMSE, and an *R*^2^ of 14.6, 31.3 nm, and 0.91, respectively, when applied to a test set that was generated using the random splitting approach, while a test set generated using the scaffold splitting strategy afforded an MAE, an RMSE, and an *R*^2^ of 24.7, 39.2 nm, and 0.86, respectively. These results are summarized in Table 3, while the regression analysis is shown in Figure 5 along with the corresponding error distributions in Figure 6.

We evaluated the performance of our proposed method against state-of-the-art ML models that have been reported in the literature. In order to perform a consistent comparative analysis and assess the generalizability of our method and of these approaches, we focused on model evaluation that utilized the scaffold splitting strategy, where possible, and we highlight the data sets that were used. Ju et al. realized a test set from the ChemFluor data set, whose MAE was 10.46 nm when applying GBRTs with the random splitting strategy.^25^ Greenman et al. verified this result by illustrating that the GBRTs can indeed achieve an MAE of approximately 10 nm on a test set of the Deep4Chem data set with random splitting, before further demonstrating that this MAE can increase to 27 nm when the scaffold splitting strategy is used.^48^ Additionally, Greenman et al. showed that the open-source Chemprop D-MPNN models with the multifidelity approach, coined ChempropMultiFidelity, can realize lower MAE values on all split types compared to GBRTs on test sets that were derived from the Deep4Chem data set. Furthermore, Joung et al. realized an RMSE of 31.6 nm using GCNNs on a test set of the Deep4Chem data set with a random splitting strategy.^26,45^ Greenman et al. demonstrated that ChempropMultiFidelity can improve the realized RMSE to 27.47 nm even with scaffold splitting using such a data set. These results suggest that ChempropMultiFidelity has a higher degree of generalizability and predictability when compared to both of the GBRT and GCNN modeling approaches, as adopted by Ju et al. and Joung et al. This makes ChempropMultiFidelity a suitable benchmark for our proposed method.

As previously mentioned, we trained our DR-CNN and GBFS models and computed the corresponding performance metrics (Table 3) using data sets identical to those used by Greenman et al., where the ChemDataExtractor-generated data set was excluded from the combined experimental data set (Table 1), and the scaffold splitting strategy was used to create the training and test sets. This ensured a rigorous evaluation of our method and enabled a like-for-like comparison. With 5-fold cross-validation, Greenman et al. achieved an MAE of 27.78 ± 5.07 nm, an RMSE of 47.13 ± 11.10 nm, and an *R*^2^ of 0.8 ± 0.07 using the ChempropMultiFidelity model on the test set,^48^ while our proposed multifidelity method realized errors in energy which, once converted into wavelength, afforded an MAE of 24.91 ± 0.64 nm, an RMSE of 39.33 ± 0.85 nm, and an *R*^2^ of 0.85 ± 0.01 using the same test set. Our results are therefore comparable to those obtained by Greenman et al. when accounting for their uncertainties, whose method is reported to outperform both the GBRTs and GCNNs. In terms of average performance, however, our modeling approach demonstrates superiority, achieving a lower mean across the three performance metrics. Our proposed method exhibits significantly lower standard errors across these metrics, indicating a more stable model with reduced variance in the predictions. For instance, considering one standard deviation, approximately 68% of the predictions are expected to fall within the defined uncertainty range. While the performance of both methods is statistically comparable, our advantage of having a more stable prediction is evident.

Moreover, the use of scaffold splitting meant that the ML models were less reliant on the combination of dye molecules resided in the training set and they were encouraged to incorporate learning from the effects of chemical composition, molecular structure, and solvent. These results demonstrate the generalizability of our proposed method in predicting the molecular absorption maxima in the optical spectrum. It is important to note that the combined experimental data sets widened the chemical landscape compared to the original, individual data sets by virtue of introducing a different chemical space that was represented by each data set. Consequently, we expect the magnitude of the errors in these predictions to be greater than those reported in the literature, which utilizes a subset of the combined experimental data set in Table 1.

We now take a closer examination of the performance of our model on dyes in which metals are present. In total, there are 308 dye molecules that contain metals, 281 in the training set and 27 in the test set with scaffold splitting. Further categorization shows that there are 267 post-transition metals, 39 transition metals, and 2 alkali metals. The analysis of the absolute errors in energy for the ML-based predictions of λ_max_ against the experimental measurements on these metal-containing dyes, once converted into wavelength, yielded an MAE of 58.0 nm. The minimum and maximum absolute errors of 4.3 and 167.5 nm were observed, respectively. Upon segregating the error analysis by the types of metals, we observed an MAE of 61.7 nm for dyes containing post-transition metals, while an MAE of 11.2 nm is observed for those containing transition metals. At a high level, the model appears to encounter difficulties in accurately predicting λ_max_ for dye molecules in the presence of metals, with larger errors being predominant in those containing post-transition metals. This stands to reason by using chemical intuition. However, it is crucial to emphasize that this observation does not constitute a definitive conclusion, given that the subset of metal-containing dyes constitutes a minority within the data set, comprising 281 out of 23,754 in the training set. We anticipate an improvement in the model accuracy with an increasing number of metal-containing dye molecules.

We also examined the performance of our model in terms of considering solvent effects. Thereby, the distribution of absolute errors in the ML-based predictions of λ_max_ against the experimental measurements was partitioned based on the eight most frequently occurring solvents in the training set using scaffold splitting (for details, see Supporting Information 5). The MAE ranges from the lowest value of 15.1 nm (for dimethyl sulfoxide, i.e., SMILES string, CS(C)=O) and the highest value of 32.8 nm (for ethanol, i.e., SMILES string, CCO). This does not imply that there is a more pronounced adverse impact on the predictive model for chemicals solvated in ethanol compared with dimethyl sulfoxide. Assessing the effect of these solvents on the prediction is challenging due to substantial variations in the number of observations among solvents and the absence of dye molecules solvated in a variety of solvents within the test set. A comprehensive exploration of the effects of solvents, encompassing their role as dielectric media or their role in more intricate phenomena such as the modification of a conformational ensemble, constitutes a multifaceted and nuanced subject. This topic is suggested for further exploration as a potential avenue for subsequent research.

We attributed the generalizability, demonstrated by the proposed method, to three components of our operational workflow. First, in contrast to a naive approach to utilizing feature descriptors, our GBFS workflow design identifies the subset of features that maximizes the relevance to the target variable while minimizing feature redundancy. This allows the exploration of a wide range of descriptors, where only a subset of features may be relevant to the prediction of the optical absorption peaks; one can avoid training an ML model with a convoluted list of features, thereby preventing the model from overfitting by achieving a better bias-variance trade-off. In other words, our GBFS workflow is able to capture the relationship between the exploratory features and the target variable while controlling the sensitivity of our method to small fluctuations, or noises, within the data set.

Second, the multifidelity modeling approach, where auxiliary ML models (both DR-CNN and GBFS) are trained on DFT data sets, appears to have a substantial impact on the prediction of λ_max_ values against their experimental measurements. For example, the DFR of the dyes and solvents learned via the DR-CNN workflow appears to have made the most significant contribution in the model prediction. Figure 7 shows that the largest total loss reduction is realized by the λ_max_ values that have been predicted using the DFR from the DR-CNN workflow, which was afforded by its training against the TD-DFT peak vertical excitation energy using random splitting. Meanwhile, the DFR itself that was learned from the TD-DFT calculations ranked first when scaffold splitting is used. Moreover, the second largest total loss reduction was realized by the prediction of λ_max_ values via the GBFS workflow, which was afforded by its training against sTDA-DFT absorption maxima.^23^ Both examples involve either the prediction or a learned representation of the DFT data set via one of the subworkflows. This demonstrates the effectiveness of the multifidelity modeling approach. These results suggest that the inclusion of readily available DFT data sets from the literature appears to help the ML models navigate the chemical space in search of experimental values of the optical absorption peaks. Alternatively, one can consider the multifidelity modeling method as creating a nested chemical space such that the search space is contained within a wider landscape defined by the computational data, which cannot be explored solely by relying on the limited number of experimental data.

**Figure 7:**
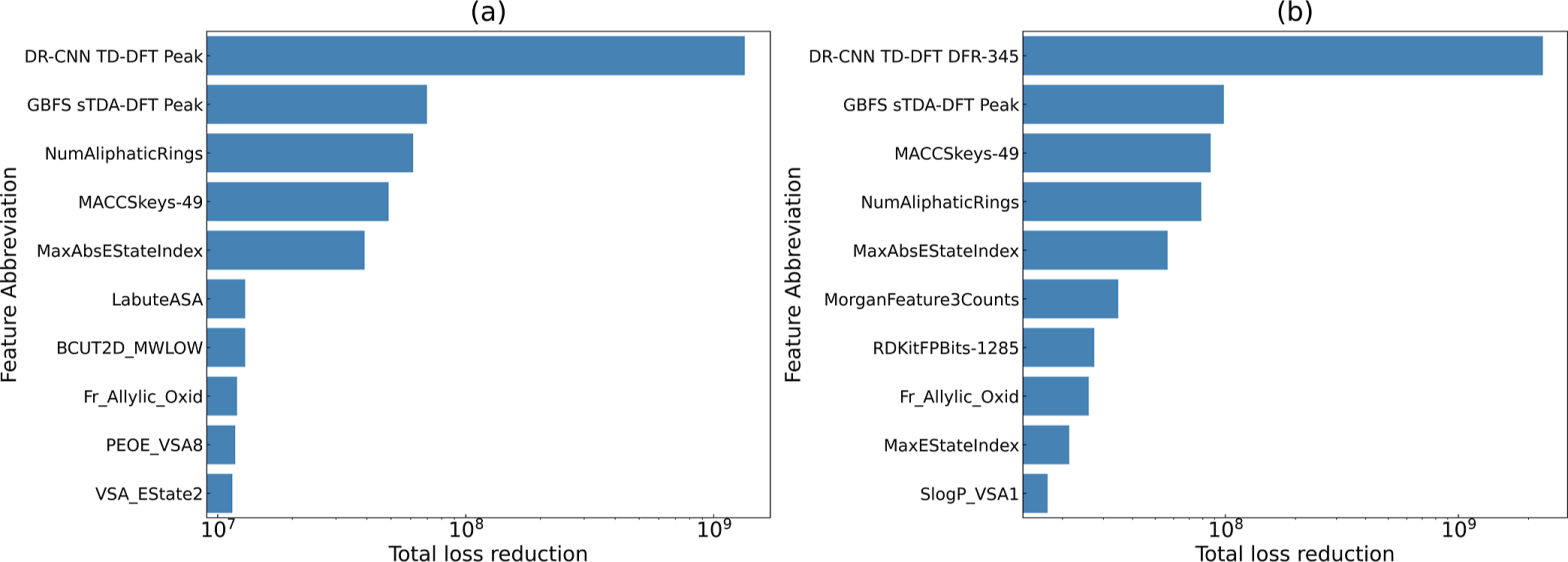
Bar chart depicting the total loss reduction realized by the top 10 features that were identified to have the most relevance to the experimental measurements of the optical absorption peak wavelength, using the (a) random and (b) scaffold splitting strategies. The corresponding feature descriptions can be found in Table 4.

Third, we highlight the important contribution that was made by the DR-CNN workflow in the final prediction of λ_max_ values against their experimental measurements. DR-CNN is able to process and learn from the feature matrices through its deep architecture and identify complex relationships between the features derived exclusively from SMILES strings and λ_max_ values. It should also be noted that the ML models that were trained on the DFT data sets can be leveraged to make separate predictions, as opposed to carrying out more costly DFT calculations for novel chemical molecules.

We note that our proposed method is not making a direct comparison between vertical transition energies, which are calculated using quantum chemical methods, and experimental absorption maxima. It is well established that these two quantities differ in their definitions. Large discrepancies can arise due to a shift between equilibrium positions of potential energy surfaces, for instance, when a harmonic model predicts the vertical transition energy as the centroid of a convolution of a Poisson distribution in the absence of Duschinsky rotation.^81−83^ Furthermore, it is essential to note that spectral shapes are determined by the vibrational envelope and can experience broadening effects, among other factors, making direct comparison or benchmarking between the two quantities not necessarily valid. Our objective is to explore whether there is additional information or underpinning relationships that can be captured by considering both pieces of information. The results indicate a considerable information gain (or reduction in loss) when vertical transition energies are taken into account by the predictive model, as illustrated in Figure 7. This suggests that despite the known disparities or deviations between the two quantities, the ML algorithm is able to capture valuable information or relationships between them, resulting in lower loss during model training.

Furthermore, it is necessary to be conscious of conformational effects in the context of spectroscopy and molecular chemistry.^84^ These effects manifest as variations in the atomic arrangement within a molecule, stemming from different spatial orientations or conformations. While spectroscopic data can offer insights into these conformational effects by representing a Boltzmann average of all conformers present in a solution, SMILES strings lack the capacity to consider such variations. Given that this study does not distinguish between cases involving single or multiple conformations in the experimental measurements, nor does it address potential modifications to the conformational ensemble due to interactions with the environment (i.e., the solvent), it is imperative to consider these factors when citing or referencing the methodology and the findings presented herein. This is pertinent not only to this study but also to other research endeavors employing SMILES representation.

### 3.3 Application of ML Models across Chemical Space

The results in Figures 4–6 and Table 3 present predictions of λ_max_ values against experimental measurements and DFT calculations, with promising statistical figures of merit on both accounts. Nonetheless, it is important to validate these results by considering how these predictions fare across the diverse range of optically active chemicals rather than simply demonstrating their collective statistical quality in an anonymized form.

Indeed, there are well-known relationships between optical absorption wavelengths and the molecular structure of organic compound.^14,85^ Thus, it would be natural to test how well these predictions of λ_*max*_ values fare against illustrative types of organic chemicals. For example, one might wish to consider if the predictions are more or less successful when a molecule has fused rings or not or if it contains many rings or rings that feature certain heteroatoms. Does the quality of the predictions vary as a function of the number of π-conjugated bonds in the molecule, the overall size of the molecule, or the length of the π-bridge in a donor–π–acceptor molecule?

**Figure 8:**
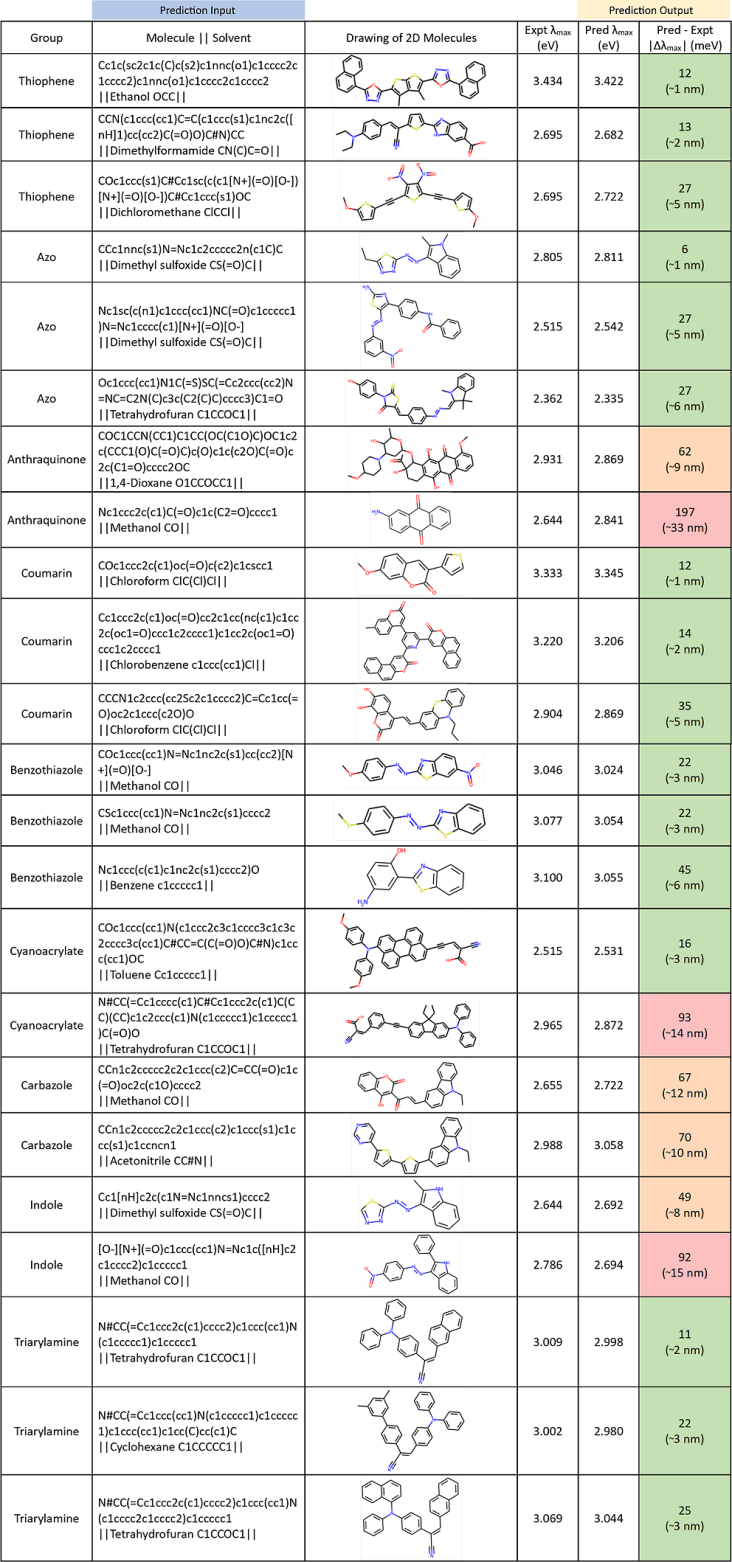
Examples of input molecules and the corresponding prediction of λ_max_ values against their experimental measurements. The input to the prediction is exclusively the SMILES string of the subject molecule, unless the user wishes to include solvent information for which its SMILES string will then be given. The output is the predicted λ_max_ value of the subject compound. We considered unseen chemical molecules from a list of nine chemical classes that are commonly found in optically absorbing organic molecules. The absolute difference between the predicted values and the experimental measurements (|Δλ_max_|) are color-coded in green (0–45 meV), amber (46–90 meV), or red (≥91 meV).

Such chemical classifications for optically active molecules on the ChemDataExtractor-generated data set of experimentally measured λ_max_ values have been made by Flanagan and Cole.^85^ They assembled a list encompassing some of the most common chemical groups that have been identified in optically absorbing organic dyes across multiple domains. The presence or absence of a functional group in a molecule is ascertained through SMARTS (SMILES Arbitrary Target Specification) pattern matching. Subsequently, a hierarchical fingerprint scheme is used to categorize similar functional molecular fragments along with illustrative examples of molecules that span across this diversity of chemical space. Thus, it seemed quite natural to apply our multifidelity ML model to the SMILES strings of the molecules that they illustrated, to test the efficacy of our predictions of λ_max_ across the rich set of optically active chemicals that can exist. Among the list of chemical classes that they examined, we chose unseen candidates with associated solvent information for analysis using our proposed methodology. The results are shown in Figure 8, where the analysis considered nine classes that are commonly found in optically absorbing organic molecules. The absolute difference of each predicted λ_max_ value from its experimental measurement is color-coded according to the classifications: green (0–45 meV), amber (46–90 meV), or red (≥91 meV).

We observe relatively higher discrepancies in three classes of chemicals listed in Figure 8, namely, anthraquinones, carbazoles, and indolines.

Poor predictions are noted for both case studies of anthraquinone molecules. The smaller one of these molecules exhibits pseudosymmetry, i.e., it would have D_2*h*_ point group symmetry if its NH_2_ group was absent; this will compromise any “push–pull” π-conjugated character that is typically associated with optically absorbing molecules. The larger anthraquinone molecule displays a minimal level of π-conjugation beyond its parent anthraquinoidal moiety; indeed, its other moiety is predominantly saturated, which will preclude intramolecular charge transfer (ICT) and thus deter the optical absorption properties of the overarching molecule. In both cases, these π-conjugated perturbations deviate from the typical π-conjugated characteristics that are observed in the other molecules shown in Figure 8; such perturbations hinder the prediction accuracy of the model for these molecules.

Another type of π-conjugated perturbation may also be responsible for the poor prediction accuracy of the second cyanoacrylate listed in Figure 8. The π-conjugated backbone of this molecule contains an alkynyl group; while this features π-bonding, the presence of two mutually perpendicular p-orbitals within its triple bond will complicate its ICT characteristics and, thus, the ability to predict the optically absorbing properties of this molecule. While there exist two alkynyl groups in one of the other molecules in Figure 8 (the third thiophene in the list), they do not lie on the only π-conjugated backbone of the molecule in contrast to this cyanoacrylate molecule, so their impact on the predicted optical absorption properties will likely be of less significance.

The poor predictive performance of the indole- and carbazole-based molecules appears to be systematic. On the one hand, this chemical trend is not surprising since carbazoles are based on the indole structure: the second benzene ring of the carbazole has typically been created through chemical substitution at the 2,3 positions of an indole structure. On the other hand, the rationale for large discrepancies between predicted and experimental values of λ_max_ for these molecules is less clear. For example, the molecular structures of the first azo and first indole chemicals listed in Figure 8 are almost identical, and yet, the prediction for this azo compound matches well to that of its optical absorption properties from the experiment, while those of the indole do not. Both compounds have been solvated in the same solvent, dimethyl sulfoxide (DMSO). One key chemical difference between these two compounds is that the nitrogen atom on the one classed as an indole exhibits a terminal hydrogen atom, while the hydrogen atom of the N on the indole ring of its comparative azo molecule has already been substituted by a methyl group. The indole will be inclined to deprotonate when solvated in DMSO, and the predictions will not capture this solvent effect or model well this tendency toward protonation, while the experimental λ_max_ value will implicitly capture this effect. The N atom of the ring in the other indole listed in Figure 8 also carries a terminal hydrogen, although its experimental λ_max_ value was captured by using methanol as the solvent. The rationale behind the significant λ_max_ differences between the prediction and experiment for the two carbazoles listed in Figure 8 is less clear.

## 4 Conclusions

This study has demonstrated the success of a method that trains two distinct types of ML workflows, in conjunction, to predict the peak optical absorption wavelength, λ_max_, that one would normally have to obtain experimentally using UV–vis spectroscopy. One of the subworkflows involves the use of deep residual convolutional neural networks, which are trained to generate deep feature representations of dye molecules and solvents; the other incorporates a gradient boosting algorithm with additional processes to identify a subset of features that affords minimal feature redundancy and maximal relevance to the target variable from a comprehensive list of descriptor features. We adopt a multifidelity approach, where auxiliary models are trained on DFT calculations. For a given SMILES string, the auxiliary models generate DFT-aware feature representations, which are subsequently concatenated with the experiment-aware feature representations that are generated by the models trained on experimental measurements. The concatenated features are subsequently used to make the final prediction of the peak optical wavelengths using a Bayesian-optimized gradient-boosting machine. The proposed method is benchmarked against state-of-the-art ML methods that have been previously reported in the scientific literature using a combination of open-source experimental data sets and the scaffold splitting strategy using the Bemis–Murcko framework. Finally, the application of our ML models to a diverse range of optically active chemicals is exemplified. This illustrates their potential for practical applications either to corroborate experimental measurements of λ_max_ values or to serve as alternatives to the computationally intensive DFT computations.

## Data Availability

We have made the code scripts used in this study available at https://github.com/Songyosk/UVVIS and https://github.com/Songyosk/GBFS4MPPML.

## Supporting Information Available

Bayesian optimization and the acquisition schemes used in this study, pseudocode for Bayesian optimization, regression analysis of the ML-based prediction of the vertical excitation energies against the DFT-based calculations, regression analysis of the ML-based prediction of the experimental optical absorption peak of unseen chemical dyes against their experimental measurements, and distributions of the absolute errors of the ML-based prediction of optical absorption peak against the experimental measurements, partitioned by solvent types (PDF)

## References

[ref1] Erb T.; Zhokhavets U.; Gobsch G.; Raleva S.; Stühn B.; Schilinsky P.; Waldauf C.; Brabec C. J. Correlation Between Structural and Optical Properties of Composite Polymer/Fullerene Films for Organic Solar Cells. Adv. Funct. Mater. 2005, 15, 1193–1196. 10.1002/adfm.200400521.

[ref2] Ball J. M.; Stranks S. D.; Hörantner M. T.; Hüttner S.; Zhang W.; Crossland E. J. W.; Ramirez I.; Riede M.; Johnston M. B.; Friend R. H.; Snaith H. J. Optical Properties and Limiting Photocurrent of Thin-Film Perovskite Solar Cells. Energy Environ. Sci. 2015, 8, 602–609. 10.1039/C4EE03224A.

[ref3] Girotto C.; Moia D.; Rand B. P.; Heremans P. High-Performance Organic Solar Cells With Spray-Coated Hole-Transport and Active Layers. Adv. Funct. Mater. 2011, 21, 64–72. 10.1002/adfm.201001562.

[ref4] Moia D.; Giovannitti A.; Szumska A. A.; Maria I. P.; Rezasoltani E.; Sachs M.; Schnurr M.; Barnes P. R.; McCulloch I.; Nelson J. Design and Evaluation of Conjugated Polymers With Polar Side Chains as Electrode Materials for Electrochemical Energy Storage in Aqueous Electrolytes. Energy Environ. Sci. 2019, 12, 1349–1357. 10.1039/C8EE03518K.

[ref5] Gracia R.; Mecerreyes D. Polymers With Redox Properties: Materials for Batteries, Biosensors and More. Polym. Chem. 2013, 4, 2206–2214. 10.1039/c3py21118e.

[ref6] Szumska A. A.; Maria I. P.; Flagg L. Q.; Savva A.; Surgailis J.; Paulsen B. D.; Moia D.; Chen X.; Griggs S.; Mefford J. T.; Rashid R. B.; Marks A.; Inal S.; Ginger D. S.; Giovannitti A.; Nelson J. Reversible Electrochemical Charging of n-Type Conjugated Polymer Electrodes in Aqueous Electrolytes. J. Am. Chem. Soc. 2021, 143, 14795–14805. 10.1021/jacs.1c06713. 34469688 PMC8447255

[ref7] Pollice R.; Friederich P.; Lavigne C.; Gomes G. d. P.; Aspuru-Guzik A. Organic molecules with inverted gaps between first excited singlet and triplet states and appreciable fluorescence rates. Matter 2021, 4, 1654–1682. 10.1016/j.matt.2021.02.017.

[ref8] Stuke A.; Kunkel C.; Golze D.; Todorović M.; Margraf J. T.; Reuter K.; Rinke P.; Oberhofer H. Atomic structures and orbital energies of 61,489 crystal-forming organic molecules. Sci. Data 2020, 7, 58 10.1038/s41597-020-0385-y. 32071311 PMC7029047

[ref9] Omar Ö. H.; Nematiaram T.; Troisi A.; Padula D. Organic materials repurposing, a data set for theoretical predictions of new applications for existing compounds. Sci. Data 2022, 9, 54 10.1038/s41597-022-01142-7. 35165288 PMC8844419

[ref10] Laurent A. D.; Adamo C.; Jacquemin D. Dye Chemistry With Time-Dependent Density Functional Theory. Phys. Chem. Chem. Phys. 2014, 16, 14334–14356. 10.1039/C3CP55336A. 24548975

[ref11] Pepe G.; Cole J. M.; Waddell P. G.; McKechnie S. Molecular Engineering of Cyanine Dyes to Design a Panchromatic Response in Co-Sensitized Dye-Sensitized Solar Cells. Mol. Syst. Des. Eng. 2016, 1 (1), 86–98. 10.1039/C6ME00014B.

[ref12] Schröder F. A. Y. N.; Cole J. M.; Waddell P. G.; McKechnie S. Transforming Benzophenoxazine Laser Dyes into Chromophores for Dye-Sensitized Solar Cells: A Molecular Engineering Approach. Adv. Energy Mater. 2015, 5 (9), 1401728 10.1002/aenm.201401728.

[ref13] Bayliss S. L.; Cole J. M.; Waddell P. G.; McKechnie S.; Liu X. Predicting Solar-Cell Dyes for Cosensitization. J. Phys. Chem. C 2014, 118, 14082–14090. 10.1021/jp501159g.

[ref14] Liu X.; Xu Z.; Cole J. M. Molecular Design of UV–vis Absorption and Emission Properties in Organic Fluorophores: Toward Larger Bathochromic Shifts, Enhanced Molar Extinction Coefficients, and Greater Stokes Shifts. J. Phys. Chem. C 2013, 117, 16584–16595. 10.1021/jp404170w.

[ref15] Liu X.; Cole J. M.; Low K. S. Solvent Effects on the UV–vis Absorption and Emission of Optoelectronic Coumarins: a Comparison of Three Empirical Solvatochromic Models. J. Phys. Chem. C 2013, 117, 14731–14741. 10.1021/jp310397z.

[ref16] Jung G.; Jung S. G.; Cole J. M. Automatic Materials Characterization From Infrared Spectra Using Convolutional Neural Networks. Chem. Sci. 2023, 14, 3600–3609. 10.1039/D2SC05892H. 37006683 PMC10055241

[ref17] Jung S. G.; Jung G.; Cole J. M. Gradient Boosted and Statistical Feature Selection Workflow for Materials Property Predictions. J. Chem. Phys. 2023, 159 (19), 194106 10.1063/5.0171540. 37971034

[ref18] Jung S. G.; Jung G.; Cole J. M. Automatic Prediction of Band Gaps of Inorganic Materials Using a Gradient Boosted and Statistical Feature Selection Workflow. J. Chem. Inf. Model. 2024, 64 (4), 1187–1200. 10.1021/acs.jcim.3c01897. 38320103 PMC10900294

[ref19] Dral P. O.; Barbatti M. Molecular Excited States Through a Machine Learning Lens. Nat. Rev. Chem 2021, 5, 388–405. 10.1038/s41570-021-00278-1. 37118026

[ref20] Westermayr J.; Marquetand P. Machine Learning for Electronically Excited States of Molecules. Chem. Rev. 2021, 121, 9873–9926. 10.1021/acs.chemrev.0c00749. 33211478 PMC8391943

[ref21] Chen C.-H.; Tanaka K.; Funatsu K. Random forest approach to QSPR study of fluorescence properties combining quantum chemical descriptors and solvent conditions. J. Fluoresc. 2018, 28, 695–706. 10.1007/s10895-018-2233-4. 29680928

[ref22] Taniguchi M.; Lindsey J. S. Database of Absorption and Fluorescence Spectra of >300 Common Compounds for use in PhotochemCAD. Photochem. Photobiol. 2018, 94, 290–327. 10.1111/php.12860. 29166537

[ref23] Beard E. J.; Sivaraman G.; Vázquez-Mayagoitia Á.; Vishwanath V.; Cole J. M. Comparative Dataset of Experimental and Computational Attributes of UV/Vis Absorption Spectra. Sci. Data 2019, 6, 307 10.1038/s41597-019-0306-0. 31804487 PMC6895184

[ref24] Venkatraman V.; Kallidanthiyil Chellappan L. An Open Access Data Set Highlighting Aggregation of Dyes on Metal Oxides. Data 2020, 5, 45 10.3390/data5020045.

[ref25] Ju C.-W.; Bai H.; Li B.; Liu R. Machine Learning Enables Highly Accurate Predictions of Photophysical Properties of Organic Fluorescent Materials: Emission Wavelengths and Quantum Yields. J. Chem. Inf. Model. 2021, 61, 1053–1065. 10.1021/acs.jcim.0c01203. 33620207

[ref26] Joung J. F.; Han M.; Jeong M.; Park S. Experimental Database of Optical Properties of Organic Compounds. Sci. Data 2020, 7, 295 10.1038/s41597-020-00634-8. 32901041 PMC7478979

[ref27] Noelle A.; Vandaele A. C.; Martin-Torres J.; Yuan C.; Rajasekhar B. N.; Fahr A.; Hartmann G. K.; Lary D.; Lee Y.-P.; Limão-Vieira P.; Locht R.; McNeill K.; Orlando J. J.; Salama F.; Wayne R. P. UV/Vis+ Photochemistry Database: Structure, Content and Applications. J. Quant. Spectrosc. Radiat. Transfer 2020, 253, 107056 10.1016/j.jqsrt.2020.107056. PMC8193824 34121770

[ref28] Venkatraman V.; Raju R.; Oikonomopoulos S. P.; Alsberg B. K. The Dye-Sensitized Solar Cell Database. J. Cheminf. 2018, 10, 18 10.1186/s13321-018-0272-0. PMC5882482 29616364

[ref29] Blum L. C.; Reymond J.-L. 970 Million Druglike Small Molecules for Virtual Screening in the Chemical Universe Database GDB-13. J. Am. Chem. Soc. 2009, 131, 8732–8733. 10.1021/ja902302h. 19505099

[ref30] Montavon G.; Rupp M.; Gobre V.; Vazquez-Mayagoitia A.; Hansen K.; Tkatchenko A.; Müller K. R.; Anatole von Lilienfeld O. Machine Learning of Molecular Electronic Properties in Chemical Compound Space. New J. Phys. 2013, 15, 095003 10.1088/1367-2630/15/9/095003.

[ref31] Ruddigkeit L.; Van Deursen R.; Blum L. C.; Reymond J.-L. Enumeration of 166 Billion Organic Small Molecules in the Chemical Universe Database GDB-17. J. Chem. Inf. Model. 2012, 52, 2864–2875. 10.1021/ci300415d. 23088335

[ref32] Ramakrishnan R.; Hartmann M.; Tapavicza E.; Von Lilienfeld O. A. Electronic Spectra From TDDFT and Machine Learning in Chemical Space. J. Chem. Phys. 2015, 143, 084111 10.1063/1.4928757. 26328822

[ref33] Liang J.; Ye S.; Dai T.; Zha Z.; Gao Y.; Zhu X. QM-Symex, Update of the Qm-Sym Database With Excited State Information for 173 Kilo Molecules. Sci. Data 2020, 7, 400 10.1038/s41597-020-00746-1. 33208742 PMC7675965

[ref34] Nakata M.; Shimazaki T. PubChemQC Project: A Large-Scale First-Principles Electronic Structure Database for Data-Driven Chemistry. J. Chem. Inf. Model. 2017, 57, 1300–1308. 10.1021/acs.jcim.7b00083. 28481528

[ref35] Steinbeck C.; Han Y.; Kuhn S.; Horlacher O.; Luttmann E.; Willighagen E. The Chemistry Development Kit (Cdk): An Open-Source Java Library for Chemo-and Bioinformatics. J. Chem. Inf. Comput. Sci. 2003, 43, 493–500. 10.1021/ci025584y. 12653513 PMC4901983

[ref36] Willighagen E. L.; Mayfield J. W.; Alvarsson J.; Berg A.; Carlsson L.; Jeliazkova N.; Kuhn S.; Pluskal T.; Rojas-Chertó M.; Spjuth O.; Torrance G.; Evelo C. T.; Guha R.; Steinbeck C. The Chemistry Development Kit (CDK) V2. 0: Atom Typing, Depiction, Molecular Formulas, and Substructure Searching. J. Cheminf. 2017, 9, 33 10.1186/s13321-017-0220-4. PMC5461230 29086040

[ref37] Morgan H. L. The Generation of a Unique Machine Description for Chemical Structures-a Technique Developed at Chemical Abstracts Service. J. Chem. Doc. 1965, 5, 107–113. 10.1021/c160017a018.

[ref38] Hall L. H.; Kier L. B. Electrotopological State Indices for Atom Types: A Novel Combination of Electronic, Topological, and Valence State Information. J. Chem. Inf. Comput. Sci. 1995, 35, 1039–1045. 10.1021/ci00028a014.

[ref39] Kang B.; Seok C.; Lee J. Prediction of Molecular Electronic Transitions Using Random Forests. J. Chem. Inf. Model. 2020, 60, 5984–5994. 10.1021/acs.jcim.0c00698. 33090804

[ref40] Kim S.; Thiessen P. A.; Bolton E. E.; Chen J.; Fu G.; Gindulyte A.; Han L.; He J.; He S.; Shoemaker B. A.; Wang J.; Yu B.; Zhang J.; Bryant S. H. PubChem Substance and Compound Databases. Nucleic Acids Res. 2016, 44, D1202–D1213. 10.1093/nar/gkv951. 26400175 PMC4702940

[ref41] Schmidt M. W.; Baldridge K. K.; Boatz J. A.; Elbert S. T.; Gordon M. S.; Jensen J. H.; Koseki S.; Matsunaga N.; Nguyen K. A.; Su S.; Windus T. L.; Dupuis M.; Montgomery J. A. Jr. General Atomic and Molecular Electronic Structure System. J. Comput. Chem. 1993, 14, 1347–1363. 10.1002/jcc.540141112.

[ref42] Gordon M. S.; Schmidt M. W. Theory and Applications of Computational Chemistry; Elsevier, 2005, pp 1167–1189.

[ref43] Weininger D. SMILES A Chemical Language and Information System. 1. Introduction to Methodology and Encoding Rules. J. Chem. Inf. Comput. Sci. 1988, 28, 31–36. 10.1021/ci00057a005.

[ref44] Landrum G. RDKit: Open-Source Cheminformatics, 2016; https://www.rdkit.org, (accessed: 01 June 2022).

[ref45] Joung J. F.; Han M.; Hwang J.; Jeong M.; Choi D. H.; Park S. Deep Learning Optical Spectroscopy Based on Experimental Database: Potential Applications to Molecular Design. JACS Au 2021, 1, 427–438. 10.1021/jacsau.1c00035. 34467305 PMC8395663

[ref46] Xie T.; Grossman J. C. Crystal Graph Convolutional Neural Networks for an Accurate and Interpretable Prediction of Material Properties. Phys. Rev. Lett. 2018, 120, 145301 10.1103/PhysRevLett.120.145301. 29694125

[ref47] Coley C. W.; Barzilay R.; Green W. H.; Jaakkola T. S.; Jensen K. F. Convolutional Embedding of Attributed Molecular Graphs for Physical Property Prediction. J. Chem. Inf. Model. 2017, 57, 1757–1772. 10.1021/acs.jcim.6b00601. 28696688

[ref48] Greenman K. P.; Green W. H.; Gómez-Bombarelli R. Multi-Fidelity Prediction of Molecular Optical Peaks With Deep Learning. Chem. Sci. 2022, 13, 1152–1162. 10.1039/D1SC05677H. 35211282 PMC8790778

[ref49] Yang K.; Swanson K.; Jin W.; Coley C.; Eiden P.; Gao H.; Guzman-Perez A.; Hopper T.; Kelley B.; Mathea M.; Palmer A.; Settels V.; Jaakkola T.; Jensen K.; Barzilay R. Analyzing Learned Molecular Representations for Property Prediction. J. Chem. Inf. Model. 2019, 59, 3370–3388. 10.1021/acs.jcim.9b00237. 31361484 PMC6727618

[ref50] Chen C.; Zuo Y.; Ye W.; Li X.; Ong S. P. Learning Properties of Ordered and Disordered Materials From Multi-Fidelity Data. Nat. Comput. Sci. 2021, 1, 46–53. 10.1038/s43588-020-00002-x. 38217148

[ref51] Huang B.; Von Lilienfeld O. A. Ab initio Machine Learning in Chemical Compound Space. Chem. Rev. 2021, 121, 10001–10036. 10.1021/acs.chemrev.0c01303. 34387476 PMC8391942

[ref52] Snoek J.; Larochelle H.; Adams R. P. Practical Bayesian Optimization of Machine Learning Algorithms. In Proceedings of the 25th International Conference on Neural Information Processing Systems; Red Hook: NY, USA, 2012; Vol. 2 pp 2951–2959.

[ref53] Shahriari B.; Swersky K.; Wang Z.; Adams R. P.; De Freitas N. Taking the Human Out of the Loop: A Review of Bayesian Optimization. Proc. IEEE 2016, 104, 148–175. 10.1109/JPROC.2015.2494218.

[ref54] Ke G.; Meng Q.; Finley T.; Wang T.; Chen W.; Ma W.; Ye Q.; Liu T.-Y. LightGBM: A Highly Efficient Gradient Boosting Decision Tree. In Proceedings of the 31st International Conference on Neural Information Processing Systems, 2017; pp 3149–3157.

[ref55] Swain M. C.; Cole J. M. ChemDataExtractor A Toolkit for Automated Extraction of Chemical Information from the Scientific Literature. J. Chem. Inf. Model. 2016, 56, 1894–1904. 10.1021/acs.jcim.6b00207. 27669338

[ref56] Hirata S.; Head-Gordon M. Time-Dependent Density Functional Theory Within the Tamm-Dancoff Approximation. Chem. Phys. Lett. 1999, 314, 291–299. 10.1016/S0009-2614(99)01149-5.

[ref57] Chai J.-D.; Head-Gordon M. Systematic Optimization of Long-Range Corrected Hybrid Density Functionals. J. Chem. Phys. 2008, 128, 084106 10.1063/1.2834918. 18315032

[ref58] Neese F.; Wennmohs F.; Becker U.; Riplinger C. The ORCA Quantum Chemistry Program Package. J. Chem. Phys. 2020, 152, 224108 10.1063/5.0004608. 32534543

[ref59] Bannwarth C.; Ehlert S.; Grimme S. GFN2-xTB—An Accurate and Broadly Parametrized Self-Consistent Tight-Binding Quantum Chemical Method With Multipole Electrostatics and Density-Dependent Dispersion Contributions. J. Chem. Theory Comput. 2019, 15, 1652–1671. 10.1021/acs.jctc.8b01176. 30741547

[ref60] Becke A. D. Density-Functional Exchange-Energy Approximation With Correct Asymptotic Behavior. Phys. Rev. A 1988, 38, 3098–3100. 10.1103/PhysRevA.38.3098. 9900728

[ref61] Grimme S.; Ehrlich S.; Goerigk L. Effect of the Damping Function in Dispersion Corrected Density Functional Theory. J. Comput. Chem. 2011, 32, 1456–1465. 10.1002/jcc.21759. 21370243

[ref62] Weigend F.; Ahlrichs R. Balanced Basis Sets of Split Valence, Triple Zeta Valence and Quadruple Zeta Valence Quality for H to Rn: Design and Assessment of Accuracy. Phys. Chem. Chem. Phys. 2005, 7, 3297–3305. 10.1039/b508541a. 16240044

[ref63] Frisch M. J.; Trucks G. W.; Schlegel H. B.; Scuseria G. E.; Robb M. A.; Cheeseman J. R.; Scalmani G.; Barone V.; Mennucci B.; Petersson G. A.; Nakatsuji H.; Caricato M.; Li X.; Hratchian H. P.; Izmaylov A. F.; Bloino J.; Zheng G.; Sonnenberg J. L.; Hada M.; Ehara M.; Toyota K.; Fukuda R.; Hasegawa J.; Ishida M.; Nakajima T.; Honda Y.; Kitao O.; Nakai H.; Vreven T.; A.Montgomery J.; Peralta J. E.; Ogliaro F.; Bearpark M.; Heyd J. J.; Brothers E.; Kudin K. N.; Staroverov V. N.; Kobayashi R.; Normand J.; Raghavachari K.; Rendell A.; Burant J. C.; Iyengar S. S.; Tomasi J.; Cossi M.; Rega N.; Millam J. M.; Klene M.; Knox J. E.; Cross J. B.; Bakken V.; Adamo C.; Jaramillo J.; Gomperts R.; Stratmann R. E.; Yazyev O.; J. Austin A.; Cammi R.; Pomelli C.; Ochterski J. W.; Martin R. L.; Morokuma K.; Zakrzewski V. G.; Voth G. A.; Salvador P.; Dannenberg J. J.; Dapprich S.; Daniels A. D.; Farkas O.; Foresman J. B.; Ortiz J. V.; Cioslowski J.; Fox D. J. Gaussian 09, Revision B.01. Gaussian Inc., Wallingford CT, 2009.

[ref64] Wang A. Y.-T.; Murdock R. J.; Kauwe S. K.; Oliynyk A. O.; Gurlo A.; Brgoch J.; Persson K. A.; Sparks T. D. Machine Learning for Materials Scientists: An Introductory Guide Toward Best Practices. Chem. Mater. 2020, 32, 4954–4965. 10.1021/acs.chemmater.0c01907.

[ref65] Artrith N.; Butler K. T.; Coudert F.-X.; Han S.; Isayev O.; Jain A.; Walsh A. Best Practices in Machine Learning for Chemistry. Nat. Chem. 2021, 13, 505–508. 10.1038/s41557-021-00716-z. 34059804

[ref66] Bemis G. W.; Murcko M. A. The Properties of Known Drugs. 1. Molecular Frameworks. J. Med. Chem. 1996, 39, 2887–2893. 10.1021/jm9602928. 8709122

[ref67] Jha D.; Ward L.; Paul A.; Liao W.-k.; Choudhary A.; Wolverton C.; Agrawal A. Elemnet: Deep Learning the Chemistry of Materials From Only Elemental Composition. Sci. Rep. 2018, 8, 17593 10.1038/s41598-018-35934-y. 30514926 PMC6279928

[ref68] Durant J. L.; Leland B. A.; Henry D. R.; Nourse J. G. Reoptimization of Mdl Keys for Use in Drug Discovery. J. Chem. Inf. Comput. Sci. 2002, 42, 1273–1280. 10.1021/ci010132r. 12444722

[ref69] Ward L.; Agrawal A.; Choudhary A.; Wolverton C. A General-Purpose Machine Learning Framework for Predicting Properties of Inorganic Materials. npj Comput. Mater. 2016, 2, 16028 10.1038/npjcompumats.2016.28.

[ref70] Deml A. M.; O’Hayre R.; Wolverton C.; Stevanović V. Predicting density functional theory total energies and enthalpies of formation of metal-nonmetal compounds by linear regression. Phys. Rev. B 2016, 93, 085142 10.1103/PhysRevB.93.085142.

[ref71] Ward L.; Dunn A.; Faghaninia A.; Zimmermann N. E.; Bajaj S.; Wang Q.; Montoya J.; Chen J.; Bystrom K.; Dylla M.; Chard K.; Asta M.; Persson K. A.; Snyder G. J.; Foster I.; Jain A. Matminer An Open Source Toolkit for Materials Data Mining. Comput. Mater. Sci. 2018, 152, 60–69. 10.1016/j.commatsci.2018.05.018.

[ref72] Ong S. P.; Richards W. D.; Jain A.; Hautier G.; Kocher M.; Cholia S.; Gunter D.; Chevrier V. L.; Persson K. A.; Ceder G. Python Materials Genomics (Pymatgen): A Robust, Open-Source Python Library for Materials Analysis. Comput. Mater. Sci. 2013, 68, 314–319. 10.1016/j.commatsci.2012.10.028.

[ref73] Carhart R. E.; Smith D. H.; Venkataraghavan R. Atom Pairs as Molecular Features in Structure-Activity Studies: Definition and Applications. J. Chem. Inf. Comput. Sci. 1985, 25, 64–73. 10.1021/ci00046a002.

[ref74] He K.; Zhang X.; Ren S.; Sun J. Deep Residual Learning for Image Recognition. In Proceedings of the IEEE Conference on Computer Vision and Pattern Recognition, 2016; pp 770–778.

[ref75] Hochreiter S. The Vanishing Gradient Problem During Learning Recurrent Neural Nets and Problem Solutions. Int. J. Uncertain. Fuzziness Knowlege-Based Syst. 1998, 06, 107–116. 10.1142/s0218488598000094.

[ref76] Glorot X.; Bengio Y. Understanding the difficulty of training deep feedforward neural networks. In Proceedings of the Thirteenth International Conference on Artificial Intelligence and Statistics; Chia Laguna Resort: Sardinia, Italy, 2010; pp 249–256.

[ref77] Ioffe S.; Szegedy C. Batch Normalization: Accelerating Deep Network Training by Reducing Internal Covariate Shift. In Proceedings of the 32nd International Conference on Machine Learning: Lille, France, 2015; pp 448–456.

[ref78] Kushner H. J. A. A New Method of Locating the Maximum Point of an Arbitrary Multipeak Curve in the Presence of Noise. J. Basic Eng. 1964, 86, 97–106. 10.1115/1.3653121.

[ref79] Mockus J.; Tiesis V.; Zilinskas A. The Application of Bayesian Methods for Seeking the Extremum. Towards Glob. Optim. 1978, 2, 117–129.

[ref80] Srinivas N.; Krause A.; Kakade S.; Seeger M. Gaussian Process Optimization in the Bandit Setting: No Regret and Experimental Design. In Proceedings of the 27th International Conference on International Conference on Machine Learning, 2010; pp 1015–1022.

[ref81] Lax M. The Franck-Condon Principle and Its Application to Crystals. J. Chem. Phys. 1952, 20, 1752–1760. 10.1063/1.1700283.

[ref82] Cederbaum L. S.; Domcke W. A many-body approach to the vibrational structure in molecular electronic spectra. I. Theory. J. Chem. Phys. 1976, 64, 603–611. 10.1063/1.432250.

[ref83] Davidson E. R.; Jarzęcki A. A. Zero point corrections to vertical excitation energies. Chem. Phys. Lett. 1998, 285, 155–159. 10.1016/S0009-2614(98)00009-8.

[ref84] Zefirov N. S. The problem of conformational effects. Tetrahedron 1977, 33, 3192 10.1016/0040-4020(77)80140-3.

[ref85] Flanagan P. J.; Cole J. M. Clustering a Database of Optically Absorbing Organic Molecules via a Hierarchical Fingerprint Scheme that Categorizes Similar Functional Molecular Fragments. J. Chem. Phys. 2022, 156, 156 10.1063/5.0087603. 35459320

