# Supplementary material for: Automatic Prediction of Peak Optical Absorption Wavelengths in Molecules Using Convolutional Neural Networks
Source: J Chem Inf Model. 2024 Feb 29;64(5):1486–501. doi: 10.1021/acs.jcim.3c01792 (PMC10934802; doi:10.1021/acs.jcim.3c01792)
Supplement: Supplementary file 1 — ci3c01792_si_001.pdf [file ci3c01792_si_001.pdf]

Supporting Information

Automatic Prediction of Peak Optical Absorption Wavelengths in Molecules using Convolutional Neural Networks

Son Gyo Jung[1,2,3], Guwon Jung[1,3,4], Jacqueline M. Cole[1,2,3,*]

[1] *Cavendish Laboratory, Department of Physics, University of Cambridge, J. J. Thomson Avenue, Cambridge, CB3 0HE, UK*

[2] *ISIS Neutron and Muon Source, STFC Rutherford Appleton Laboratory, Harwell Science and Innovation Campus, Didcot, Oxfordshire, OX11 0QX, UK*

[3] *Research Complex at Harwell, Rutherford Appleton Laboratory, Harwell Science and Innovation Campus, Didcot, Oxfordshire, OX11 0FA, UK*

[4] *Scientific Computing Department, STFC Rutherford Appleton Laboratory, Harwell Science and Innovation Campus, Didcot, Oxfordshire, OX11 0QX, UK*

[*] jmc61@cam.ac.uk

SI.1 Bayesian Optimization using Gaussian Processes

In Bayesian optimization, a probabilistic surrogate model is built to approximate an objective function f, which is based on a performance metric that is to be maximized (or minimized) under three constraints: (i) the analytical expression of f and its derivatives are unknown (i.e. no closed form), (ii) f is expensive to evaluate, and (iii) the evaluations of f may result in noisy responses. In this work, the surrogate model is a Gaussian process (GP): the generalization of a Gaussian distribution to a distribution over functions that is characterized by mean and covariance (or positive definite kernel) functions, and it consists of a prior distribution that represents the prior beliefs over all possible f. The optimization algorithm sequentially refines the surrogate model following a set of observations via Bayesian posterior updating, yielding a posterior mean and variance functions that better approximate f over the space of the objective functions[1–4].

The use of GPs to construct the surrogate model stems from the fact that Gaussian distributions are self-conjugate with respect to a Gaussian likelihood function; that is, a Gaussian prior yields a Gaussian posterior when the likelihood function is also Gaussian, that is:

$$\underbrace{P(f|D_{1:t})}_{\text{GP Posterior}} \propto \overbrace{L(D_t|f)}^{\text{Likelihood}} \underbrace{P(f|D_{1:t-1})}_{\text{GP Prior}}, \tag{1}$$

where the observations are of the form $D_{1:T} = \{(\boldsymbol{\theta}_{t=1}, \phi_{t=1}), ..., (\boldsymbol{\theta}_T, \phi_T)\}$ for a total of T observations with query points $\boldsymbol{\theta}$ within the hyperparameter space and the output ϕ_T of f. Therefore, with a conjugate prior for a Gaussian likelihood function, the posterior mean and covariance functions can be computed given the set of observations[3–6].

Bayesian optimization employs an acquisition function, which uses the GP posterior, to evaluate the utility of candidate points within the hyperparameter space that may exhibit an improvement to the current best evaluation of $f(\boldsymbol{\theta})$. There exists a trade-off between exploration and exploitation, since an acquisition function wishes to 'explore' regions of high uncertainty in the posterior, while 'exploiting' known optimal regions where the posterior mean, and therefore, the objective function is expected to be high. The next query point $\boldsymbol{\theta}_{t+1}$ is realized by maximizing the acquisition function with respect to the exploration of a high-variance region and the exploitation of a high-mean region, both of which yield high acquisition values. The surrogate model is then sequentially updated after evaluating each query point, thereby producing a more informative posterior distribution[2,7–10]. Three acquisition functions, denoted by α, were employed in this

work, each corresponding to one of the following acquisition schemes: (i) Probability of Improvement (PI)[7], (ii) Expected Improvement (EI)[8,9], and (iii) Upper-Confidence-Bounds (UCB)[10]. See Supporting Information 2 (SI.2) for the pseudo-code of Bayesian optimization implemented.

Note that the acquisition strategies described assume that the objective function is based on a performance metric which is to be maximized. However, if the objective function is to be minimized, the next query point is determined by maximizing the negative of the acquisition functions or by taking the lower confidence bound.

SI.2 Pseudo-code of Bayesian optimization

Algorithm 1: Bayesian optimization with Gaussian process prior

input: objective function f, hyper-parameter space $\boldsymbol{\theta}$, acquisition functions α,

initialization points T_{init}, maximum number of evaluation T

$y_{best} \leftarrow 0$;

for $t = 1$ **to** T_{init} **do**

select $\boldsymbol{\theta}_t$ via randomly sampling;

compute exact objective function $y_t \leftarrow f(\boldsymbol{\theta}_t)$;

if $y_t > y_{best}$ **then**

$\boldsymbol{\theta}_{best} \leftarrow \boldsymbol{\theta}_t$;

$y_{best} \leftarrow y_t$;

end

end

for $t = T_{init} + 1$ **to** T **do**

build probabilistic model for f conditioned on previous observations $D_{1:t-1}$;

compute all possible true functions using Gaussian process regression;

optimise acquisition functions α independently based on the posterior distribution

and propose a candidate point for each acquisition scheme

$\boldsymbol{\theta}_{t,s} \leftarrow argmax_{\boldsymbol{\theta}}\ \alpha_s(\boldsymbol{\theta}|D_{1:t-1})$ for $s = \{PI, EI, UCB\}$;

choose next evaluation point $\boldsymbol{\theta}_t \leftarrow argmax_{\boldsymbol{\theta}}\ softmax(\mu(\boldsymbol{\theta}_{t,s}))$;

compute exact objective function $y_t \leftarrow f(\boldsymbol{\theta}_t)$;

if $y_t > y_{best}$ **then**

$\boldsymbol{\theta}_{best} \leftarrow \boldsymbol{\theta}_t$

$y_{best} \leftarrow y_t$

end

end

return $\boldsymbol{\theta}_{best}$

Three acquisition functions, denoted by α, each correspond to one of the following acquisition schemes: (i) Probability of Improvement (PI), (ii) Expected Improvement (EI), and (iii) Upper-Confidence-Bounds (UCB).

SI.3 DFT-aware auxiliary model performance

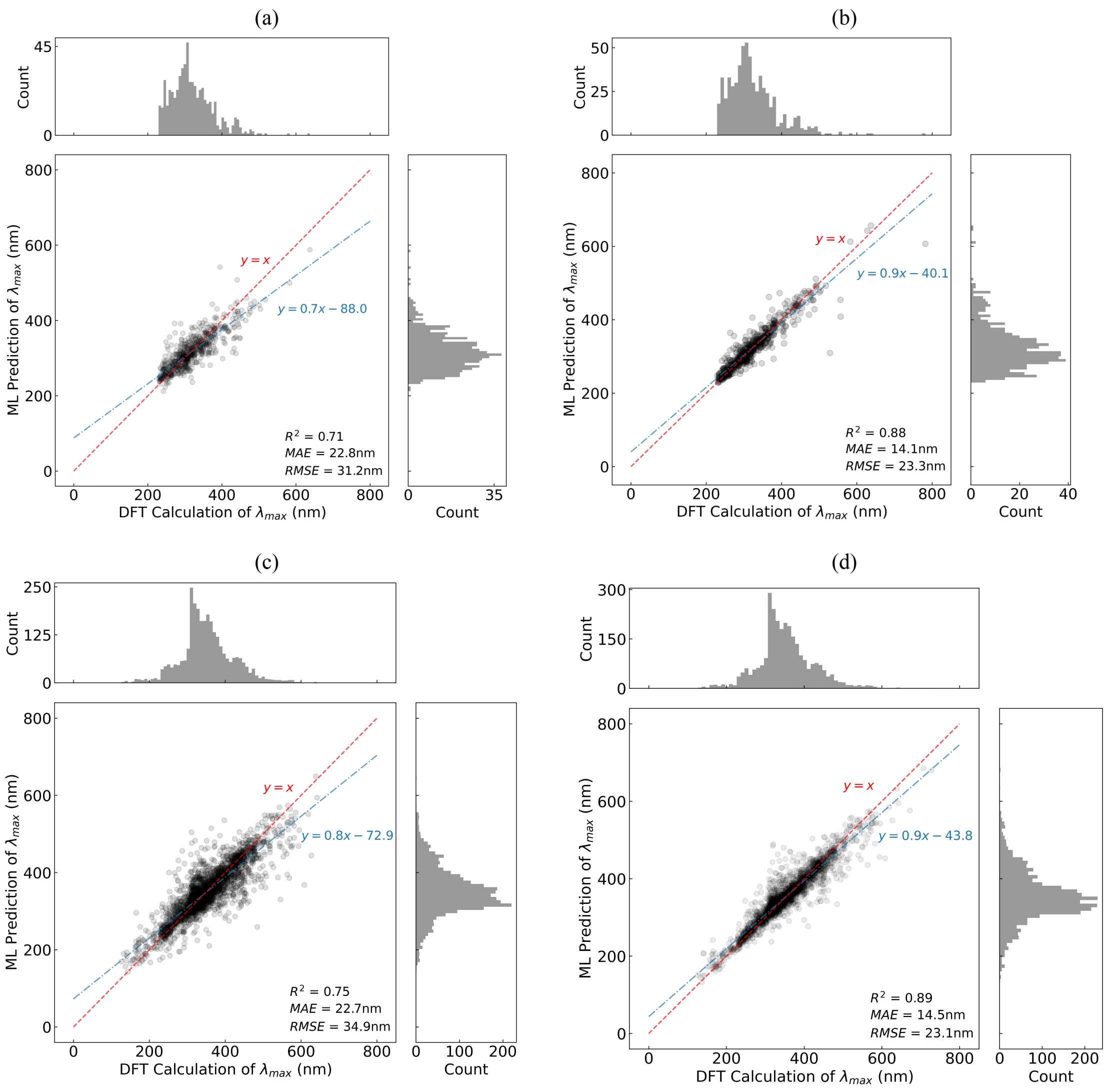

Figure S3: The regression analysis of the ML-based prediction of the vertical excitation energies, and their corresponding peak wavelengths (λ_{max}), against the DFT-based calculations. The predictions were made by Bayesian-optimized gradient boosting models that were trained on the sTDA-DFT data set via (a) the DR-CNN and (b) the GBFS sub-workflows, and on the TD-DFT data set via (c) the the DR-CNN and (d) the GBFS sub-workflows. The solid blue line is a linear fit between the DFT and ML-based predictions that was generated using ordinary least squares refinement. The dashed red line is drawn to represent the hypothetical case, where the ML-based prediction would equal the DFT-based calculations.

SI.4 Experimental-aware ML model performance

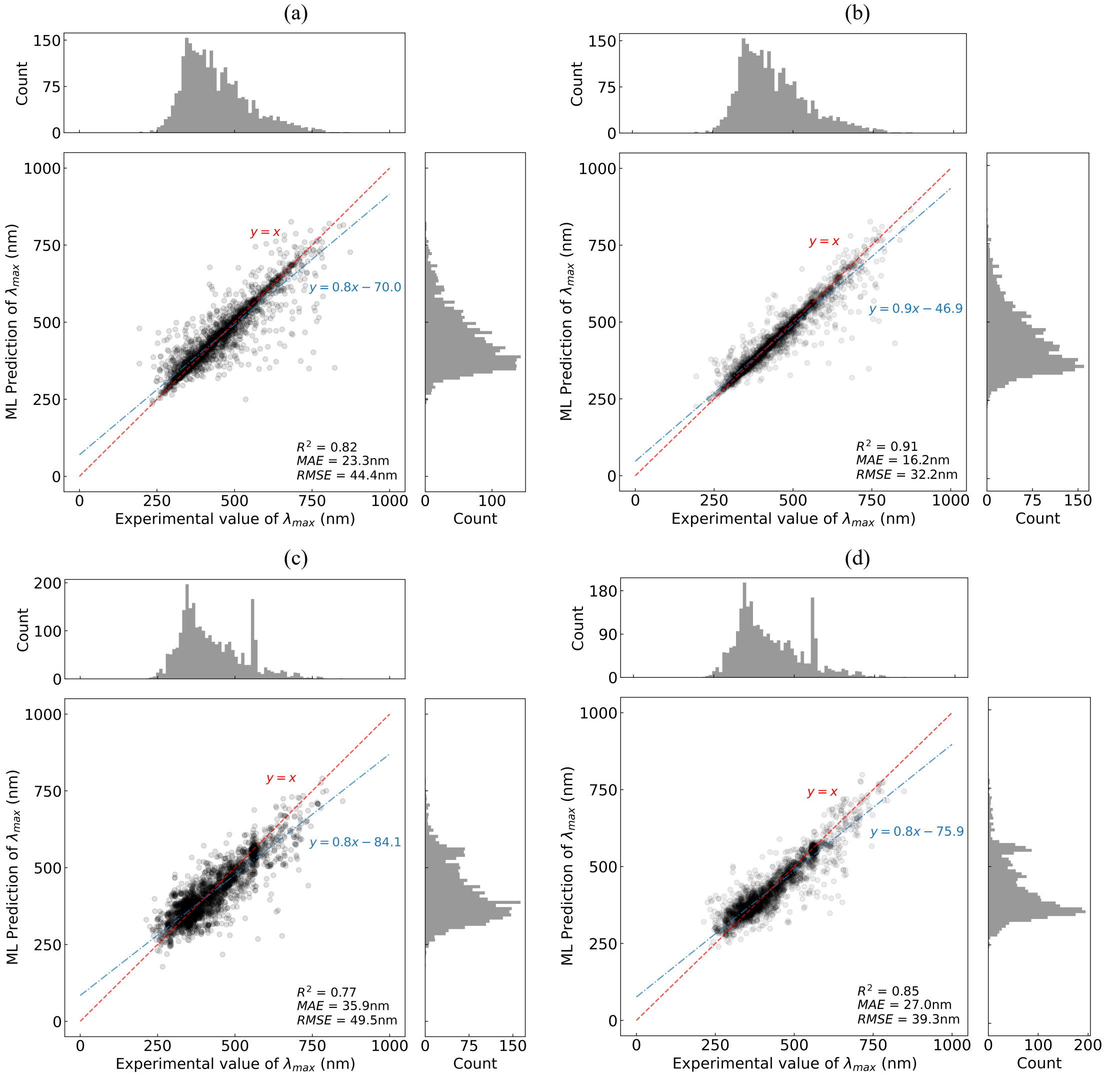

Figure S4: The regression analysis of the ML-based prediction of the experimental optical absorption peak (λ_{max}) of unseen chemical dyes against their experimental measurements. The predictions were made by Bayesian-optimized gradient boosting models that were trained using the random splitting strategy via (a) the DR-CNN and (b) the GBFS sub-workflows, and using the scaffold splitting strategy via (c) the DR-CNN and (d) the GBFS sub-workflows. The solid blue line is a linear fit between the experimental measurements and ML-based predictions that was generated using ordinary least squares refinement. The dashed red line is drawn to represent the hypothetical case, where the ML-based prediction would equal the experimental measurements.

SI.5 Error Distribution by Type of Solvents

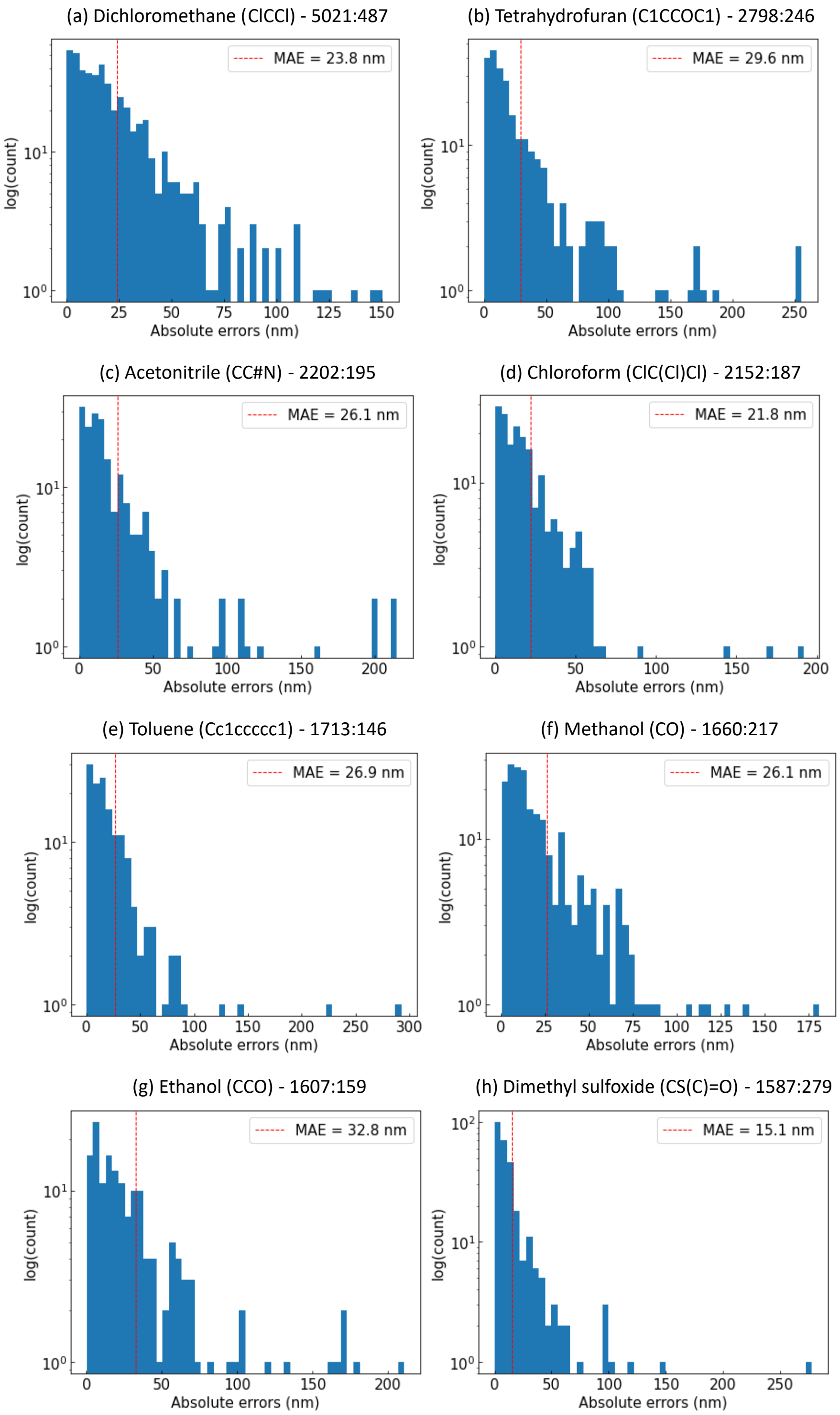

Figure S5: Distributions of the absolute errors of the ML-based prediction of λ_{max} against the experimental measurements, partitioned by solvent types. The ratio signifies the occurrence frequency of the solvent in the training and test sets with scaffold splitting, respectively.

References

[1] Snoek, J.; Larochelle, H.; Adams, R. P. Practical Bayesian Optimization of Machine Learning Algorithms. Proceedings of the 25th International Conference on Neural Information Processing Systems - Volume 2. Red Hook, NY, USA, 2012; p 2951–2959.

[2] Shahriari, B.; Swersky, K.; Wang, Z.; Adams, R. P.; De Freitas, N. Taking the Human Out of the Loop: A Review of Bayesian Optimization. *Proc. IEEE* **2015**, *104*, 148–175.

[3] Rasmussen, C. E. Gaussian Processes in Machine Learning. Proceedings of the Summer School on Machine Learning. 2003; pp 63–71.

[4] Williams, C. K.; Rasmussen, C. E. *Gaussian Processes for Machine Learning*; MIT press Cambridge, MA, 2006; Vol. 2.

[5] Murphy, K. P. *Machine Learning: A Probabilistic Perspective*; MIT press, 2012.

[6] Tipping, M. E. *Advanced Lectures on Machine Learning*; Springer, 2003; pp 41–62.

[7] Kushner, H. J. A New Method of Locating the Maximum Point of an Arbitrary Multipeak Curve in the Presence of Noise. *J. Basic Eng.* **1964**, *86*, 97–106.

[8] Mockus, J.; Tiesis, V.; Zilinskas, A. The Application of Bayesian Methods for Seeking the Extremum. *Towards Glob. Optim.* **1978**, *2*, 117–129.

[9] Mockus, J. The Bayesian approach to global optimization. System Modeling and Optimization: Proceedings of the 10th IFIP Conference New York City, USA, August 31–September 4, 1981. 2005; pp 473–481.

[10] Srinivas, N.; Krause, A.; Kakade, S.; Seeger, M. Gaussian Process Optimization in the Bandit Setting: No Regret and Experimental Design. Proceedings of the 27th International Conference on International Conference on Machine Learning. Madison, WI, USA, 2010; p 1015–1022.
